# Dynamics of biotic resistance to plant invasions

**DOI:** 10.1002/brv.70190

**Published:** 2026-06-03

**Authors:** Christine S. Sheppard, Joshua I. Brian, Wayne Dawson, Petr Dostál, Franz Essl, Michal Gruntman, Tina Heger, Mark van Kleunen, Mialy Razanajatovo, Lena C. Reimann, Frank M. Schurr, Montserrat Vilà, Florencia A. Yannelli, Marina Golivets

**Affiliations:** ^1^ Institute of Landscape and Plant Ecology, University of Hohenheim Stuttgart 70593 Germany; ^2^ Center for Biodiversity and Integrative Taxonomy (KomBioTa), University of Hohenheim Stuttgart 70593 Germany; ^3^ Institute of Botany, Department of Ecosystem Management Climate and Biodiversity, BOKU University Gregor‐Mendel‐Straße 33 Vienna 1180 Austria; ^4^ Department of Geography King's College London London WC2B 4BG UK; ^5^ Department of Evolution, Ecology and Behaviour Institute of Infection, Veterinary, and Ecological Sciences, University of Liverpool Liverpool L69 72B UK; ^6^ Institute of Botany of the Czech Academy of Sciences Průhonice CZ‐252 43 Czech Republic; ^7^ Division of BioInvasions, Global Change and Macroecology, Department of Botany and Biodiversity Research University Vienna Rennweg 14 Vienna 1030 Austria; ^8^ School of Plant Sciences and Food Security, Faculty of Life Sciences Tel Aviv University 55 Chaim Levanon St Tel Aviv‐Yafo 6997801 Israel; ^9^ Leibniz Institute of Freshwater Ecology and Inland Fisheries (IGB) Müggelseedamm 310 Berlin 12587 Germany; ^10^ Freie Universität Berlin, Institute of Biology Königin‐Luise‐Str.1–3 Berlin 14195 Germany; ^11^ Technical University of Munich, School of Life Sciences Emil‐Ramann‐Str. 6 Freising 85354 Germany; ^12^ Leuphana University Lüneburg, Institute of Ecology Universitätsallee 1 Lüneburg 21335 Germany; ^13^ Department of Biology University of Konstanz Universitätsstrasse 10 Konstanz 78464 Germany; ^14^ Zhejiang Provincial Key laboratory of Plant Evolutionary Ecology and Conservation Taizhou University Taizhou 318000 PR China; ^15^ Zhejiang Key Laboratory for Restoration of Damaged Coastal Ecosystems School of Life Sciences, Taizhou University Taizhou Zhejiang 318000 China; ^16^ Cluster of Excellence (EXC 3099): GreenRobust ‐ Understanding robustness of plant systems from molecules to ecosystems, University of Hohenheim Stuttgart 70593 Germany; ^17^ Cluster of Excellence (EXC 3121): TERRA – Terrestrial Geo‐Biosphere Interactions in a Changing World, University of Tübingen Geschwister‐Scholl‐Platz Tübingen 72074 Germany; ^18^ Doñana Biological Station (EBD‐CSIC) Sevilla 41092 Spain; ^19^ Department of Plant Biology and Ecology University of Sevilla Sevilla 41012 Spain; ^20^ Argentine Institute for Dryland Research, CONICET and Universidad Nacional de Cuyo Av. Ruiz Leal s/n Mendoza 5500 Argentina; ^21^ Department of Community Ecology Helmholtz Centre for Environmental Research – UFZ Halle 06120 Germany

**Keywords:** alien species, antagonism, biotic resistance, diversity‐invasibility, herbivory, meta‐analysis, plant competition, plant invasion, population dynamics

## Abstract

Biotic resistance, the reduction in invasion success caused by native communities, plays an important role in the long‐term dynamics of biological invasions. A large body of empirical research on biotic resistance has accumulated since the last comprehensive review on the subject 20 years ago, enabling us to achieve a refined understanding of biotic resistance and its dynamics. Here, we aim to reshape research on biotic resistance to alien plant invasions by (*i*) synthesizing existing evidence on biotic resistance and (*ii*) exploring the so far rarely considered interplay between biotic resistance mechanisms (i.e. competition, aboveground and belowground antagonisms, and diversity–invasibility effects) and the potential eco‐evolutionary changes in biotic resistance over time. To address the first aspect, we conducted a global meta‐analysis of 240 experimental studies to assess the mechanisms by which and the extent to which biotic resistance of native communities affects the performance of alien plant species. We show that competition with native plant species, aboveground antagonism (e.g. herbivores) and diversity–invasibility effects significantly reduced alien plant performance, whereas there was no evidence for consistent effects of belowground antagonism (e.g. soil pathogens). Competition exerted the strongest biotic resistance, followed by aboveground antagonism. However, the strength of biotic resistance also depended on the alien plant performance measure considered (vegetative performance, survival, reproductive performance, or population growth). From the small set of studies that considered more than one biotic resistance mechanism, we did not detect an overall synergistic effect of combined mechanisms. The meta‐analysis results also revealed that biotic resistance first decreased with the residence time of the alien plant species but increased again after approximately 200 years. In a subset of studies directly comparing species of different origin, we did not detect a difference in biotic resistance to alien *versus* native species. To address the second aspect, we expanded the limited empirical evidence on temporal dynamics by presenting a conceptual causal network and an accompanying mathematical model to explore the eco‐evolutionary dynamics of biotic resistance mechanisms. Our conceptual and mathematical models highlight that biotic resistance is determined by both the attributes of the alien species (i.e. invasiveness) and of the recipient community (i.e. invasibility). Both factors can change over time as inter‐ and/or intraspecific selection cause changes in the composition and overall density of the native community and the alien species. As invaders evolve and the successful ones persist, biotic resistance initially decreases, then increases again due to intra‐ and interspecific adaptation of the native community. Using the findings from the comprehensive synthesis of empirical studies and our modelling approach, we highlight research avenues to better understand the temporal dynamics of biotic resistance to plant invasions, including how biotic resistance depends on multiple mechanisms and performance measures, how it may differently affect alien *versus* native species and crucially, how it changes over time.

## I. INTRODUCTION

Ecologists have long recognized that ecological communities vary in their ability to resist invasions by species from geographically distant regions. This concept is central to the biotic resistance hypothesis and is heavily rooted in coexistence theory (Elton, 1958; Levine, Adler & Yelenik, 2004), which emphasizes the role of environmental conditions and species interactions in shaping community dynamics and influencing species success (Chesson, 2000). Elton (1958) formulated the biotic resistance hypothesis, also known as the diversity–invasibility hypothesis, in the context of invasions: he suggested that high species diversity enhanced community stability, thereby preventing or limiting the establishment of incoming alien species (‘invaders’) by reducing competition‐free niche space or increasing the likelihood of regulation by resident natural enemies.

Modern coexistence theory in the framework of biological invasions predicts that species coexistence and exclusion are based on the relationship between niche differences and fitness differences between native and alien competing species (MacDougall, Gilbert & Levine, 2009). Niche differences reflect the degree to which intraspecific competition exceeds interspecific competition, while fitness differences consider species demographic rates and competitive abilities regardless of their relative abundance. Coexistence is expected if niche differences between species are greater than fitness differences (Chesson, 2000). However, competition is only a particular interaction within the same trophic level influencing biotic resistance to invasion. Therefore, Levine *et al*. (2004, p. 975) expanded the definition of biotic resistance by describing it as “the reduction in invasion success caused by the resident community”. This definition encompasses biotic interactions with resident native species within the same (i.e. competition) and different (i.e. antagonism) trophic levels that reduce the invasion success (Levine *et al*., 2004). Biotic resistance is opposed to biotic facilitation by positive interactions (e.g. pollinators, seed dispersers, mycorrhiza, or plants providing beneficial microclimate) with native species. What makes predictions of interactions of native communities and alien plants even more complex is that the effects of various mechanisms of biotic resistance (and possibly, facilitation) may not act in additive ways (i.e. the effect of multiple combined mechanisms is the sum of the effects of each individual mechanism). Instead, the combined mechanisms may act synergistically (i.e. the effect is greater than expected, which would facilitate suppression of invaders), antagonistically (i.e. the effect is smaller, which would reduce suppression of invaders), or reversal (i.e. biotic resistance changes to biotic facilitation or *vice versa*).

In this paper, we define biotic resistance as the reduction in invader performance (i.e. vegetative performance, survival, reproductive performance, or population growth) due to the presence of the native community, resulting from processes that integrate both invasiveness and invasibility (see also Godoy, 2019), and involving the various mechanisms of competition, aboveground and belowground antagonisms, and diversity–invasibility effects as defined in Table 1. Invasiveness refers to the intrinsic attributes of an alien species to invade, mainly depending on plant traits (such as fast growth rate or high competitive ability), while invasibility refers to the attributes of the recipient community and thus its vulnerability to be invaded. Invasibility is referring to both the abiotic (e.g. climatic conditions) and biotic factors (e.g. interactions with native species) suitable for the alien species to establish and persist in the community where it has been introduced. The various mechanisms of biotic resistance are also relevant to the arrival of native species and general community assembly processes (e.g. range expansions or reintroductions of locally extinct native species), but these mechanisms likely act differently on alien *versus* native species due to the lack of shared co‐evolutionary history of alien species with the native community (Buckley & Catford, 2016; Sheppard & Schurr, 2019). From an applied perspective, to reduce the impacts of invasive alien species and increase restoration success, biotic resistance is particularly relevant in the context of alien plant invasions.

**Table 1:** Definitions of mechanisms of biotic resistance to alien^1^ plant invasions and plant performance measures as used in the meta‐analysis. ^1^ = We acknowledge and respect the arguments made against using terms such as “alien” in invasion biology as they may carry negative social and political connotations (e.g. Soto *et al.*, 2024; Rice *et al.*, 2025), but retain these terms to keep terminology consistent with the invasion framework (e.g. Blackburn *et al.*, 2011) as well as major international and political bodies such as the Convention of Biological Diversity (CBD), The Intergovernmental Science‐Policy Platform on Biodiversity and Ecosystem Services (IPBES) and the European Commission. ^2^ = If various performance measures were reported in a paper within one category, only one was used; the order given in the table indicates the order of preference. In particular, population‐level measures were preferred over individual measures, with population referring to a group of individuals of a species co‐occurring at the same time, either in a pot or plot. See Fig. S1 for an overview of performance measures in the included studies.

Term	Definition	Experimental approaches and measurements^2^
*Biotic resistance mechanisms*		
Competition 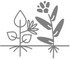	Resistance provided by the presence of a native plant species or plant community (e.g. *via* niche overlap or high competitive ability; includes both competition for abiotic resources and allelopathy)	Seeding/transplanting focal species into disturbed *vs*. undisturbed plots; growing focal species in pots with *vs*. without competitors
Aboveground antagonism 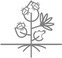	Resistance provided by the removal or degradation of plant tissue by aboveground consumers (e.g. mammalian grazers, insect herbivores, foliar fungal pathogens)	Exclusion of antagonists through enclosures or pesticide application *vs*. presence of antagonists; addition *vs*. no addition of specific antagonists to focal species
Belowground antagonism 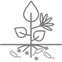	Resistance provided by the removal or degradation of plant tissue by belowground consumers (e.g. insect herbivores, nematodes, root fungal or bacterial pathogens)	Experimental removal or application of antagonists from or to focal species; plant–soil feedback experiments growing focal species in conditioned (i.e. soil that has previously been occupied by the plant species of interest) *vs*. sterile soil
Diversity–invasibility 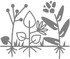	Resistance provided by native communities with high levels of taxonomic, functional or phylogenetic diversity	Seeding/transplanting focal species into pots/plots of low‐diversity *vs*. high‐diversity communities
*Plant performance measures*		
Vegetative performance 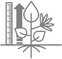	Measures of growth investment or output	Population biomass, population cover, population stand density or population abundance, individual biomass, individual growth traits
Survival 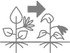	Number of individuals surviving at one or more life stages	Adult survival or mortality, seedling establishment, seed germination, seed survival or mortality
Reproductive performance 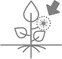	Measures of reproductive investment or output	Reproductive output (e.g. seeds, fruits, mass of reproductive structures) at the population level, reproductive output at the individual level
Population growth 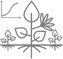	Population growth rate (ratio of population size at time *T + 1* to *T*)	Lambda, other

Since Elton formulated the biotic resistance hypothesis, numerous observational and experimental studies have investigated biotic resistance in plant communities. Part of this body of empirical research was synthesized in a pivotal meta‐analysis by Levine *et al*. (2004), which examined 52 biotic resistance studies. Across the examined studies, three key properties of communities emerged as potential mechanisms of biotic resistance: stability resulting from species diversity, competition from dominant native species, and herbivory negatively affecting invaders, though these biotic interactions rarely led to complete suppression of the invaders. In the last 20 years, over 200 additional studies were published on biotic resistance, enabling us to now address questions left unanswered in the meta‐analysis by Levine *et al*. (2004). For example, we can develop a more refined understanding of which biotic resistance mechanisms limit which plant performance aspects, whether multiple mechanisms might interact non‐additively, and to what degree it is distinct from general community assembly theory. Furthermore, while most research reviewed previously focused on plant competition (e.g. 70% of the studies in Levine *et al*., 2004), there has been an increase in the study of other biotic resistance mechanisms such as herbivory (Agrawal & Maron, 2022) and further antagonistic interactions towards alien plants such as by soil fungal communities (Inderjit & van der Putten, 2010). Also, invasion success (and conversely, biotic resistance to invasion), likely depends on the type of performance measure (Sheppard & Lüpke, 2024). While most plant invasion studies focus on biomass‐related measures, other demographic variables such as survival and reproductive performance are increasingly studied (Sheppard & Lüpke, 2024). Beyond individual‐level performance, population‐level measures, and ultimately population growth rates, provide more relevant measures directly related to invasion success. Thus, a better understanding of biotic resistance can now be achieved through a targeted meta‐analysis that leverages the wealth of studies accumulated over the past 20 years, with careful selection and exploration of the many varied approaches used.

One key aspect of biotic resistance that remains largely unexplored is how biotic resistance may change over time since the initial invasion and what role eco‐evolutionary responses in both the invader and the native community play in such dynamics. As invaders increase in density, negative interactions may cause declines in native species, possibly leading to local extinctions. Alternatively, native species may initially respond *via* phenotypic plasticity (e.g. Phillips & Leger, 2015), and in the longer term, this may facilitate genetic adaptation (Berthon, 2015). The imposed selection pressure might, with sufficient standing genetic variation, select for native genotypes that can maintain higher fitness in the presence of the invader, with coexistence as an alternative endpoint (Callaway *et al*., 2005; Lankau, 2011). Indeed, a previous meta‐analysis found that natives that had experienced invasive plants performed better than naïve native plants (Oduor, 2013). At the community level, the invader might allow only the more competitive native species to persist. The invader's impact on some native species might also release others from strong population regulation, resulting in shifts in competition or facilitation and ultimately community composition (Godoy, 2019). Understanding that invaded communities are dynamic systems with eco‐evolutionary responses and counter‐responses changing over time (Lankau, 2012; Sheppard & Schurr, 2019; Germain, Srivastava & Angert, 2020; Dostál, 2024) is important for managing alien species and the communities they invade. Despite this, most studies on biotic resistance are short‐term, mostly focusing on a single alien species, and thus cannot account for eco‐evolutionary changes in either native species or invaders that may influence the strength of biotic resistance through time. Since the lack of studies means this cannot be addressed in our meta‐analysis, we developed a conceptual and corresponding mathematical model to highlight how biotic resistance to multiple invaders might change due to eco‐evolutionary dynamics that ensue after an alien species has successfully invaded a community.

This review aims to reshape research on biotic resistance by synthesizing existing evidence, exploring the research gaps, and conceptually advancing our understanding of biotic resistance. We explore the mechanisms of biotic resistance by native communities and the extent to which it affects the performance of alien plant species, as well as how biotic resistance changes over time. Specifically, we addressed five research questions: (*1*) do native species consistently exert biotic resistance (i.e. through competition, aboveground and belowground antagonisms, and diversity–invasibility effects) to alien plant species, and if so, which biotic resistance mechanisms exert the strongest effects, and how do these effects vary depending on alien plant performance measures (i.e. vegetative performance, survival, reproductive performance, and population growth)?; (*2*) do multiple biotic resistance mechanisms have additive effects, or do they interact resulting in synergistic, antagonistic, or reversal effects on alien plants?; (*3*) does the strength of biotic resistance vary with the residence time of the alien species, the experimental methods, or the study system?; (*4*) does biotic resistance affect native plant species differently than alien invaders?; (*5*) how does biotic resistance trigger and respond to eco‐evolutionary dynamics in invaded communities?

To answer these questions, we take two complementary approaches. We address questions 1–4 with a meta‐analysis of 240 studies (methods in Section II and results in Section III), updating and extending the findings of Levine *et al*. (2004). To answer question 5, we model the eco‐evolutionary dynamics of biotic resistance (Section IV). We first develop a conceptual causal network graph (see Heger, 2022) of biotic resistance dynamics and highlight causal links between individual processes with examples from the biological invasion literature. We then provide a mathematical implementation of this causal network to demonstrate how these causal links lead to shifts in the strength of biotic resistance through time. Together, these complementary approaches highlight the currently limited understanding of how interacting mechanisms of biotic resistance influence alien plant performance and how biotic resistance changes over time through inter‐ and intraspecific selection in alien and native species.

## II. META‐ANALYSIS: METHODS

### (1) Literature search, study selection and data extraction

We searched for published studies on biotic resistance using the search platforms Scopus and Web of Science and the keyword string: (‘biotic resistance’ OR ‘invasion resistance’ OR ‘community invasibility’) AND plant* AND (alien OR introduced OR invas* OR invad* OR exotic OR non‐indigenous OR non‐native OR native). Given the different coverage of journals, both databases were used to reduce bias. This first search returned 685 records for Scopus (08/02/2024) and 1161 for Web of Science (07/03/2024). We also searched all studies that cited Levine *et al*. (2004), which returned 1096 in Scopus (08/02/2024) and 1072 in Web of Science (07/03/2024). Out of 4014 records in total, the two searches resulted in 1528 and 1308 non‐duplicate studies in Scopus and Web of Science, respectively. We also considered the 44 studies included in Levine *et al*. (2004). Overall, this search resulted in 1957 non‐duplicate studies (peer‐reviewed scientific articles) (Fig. 1).

**Fig. 1:**
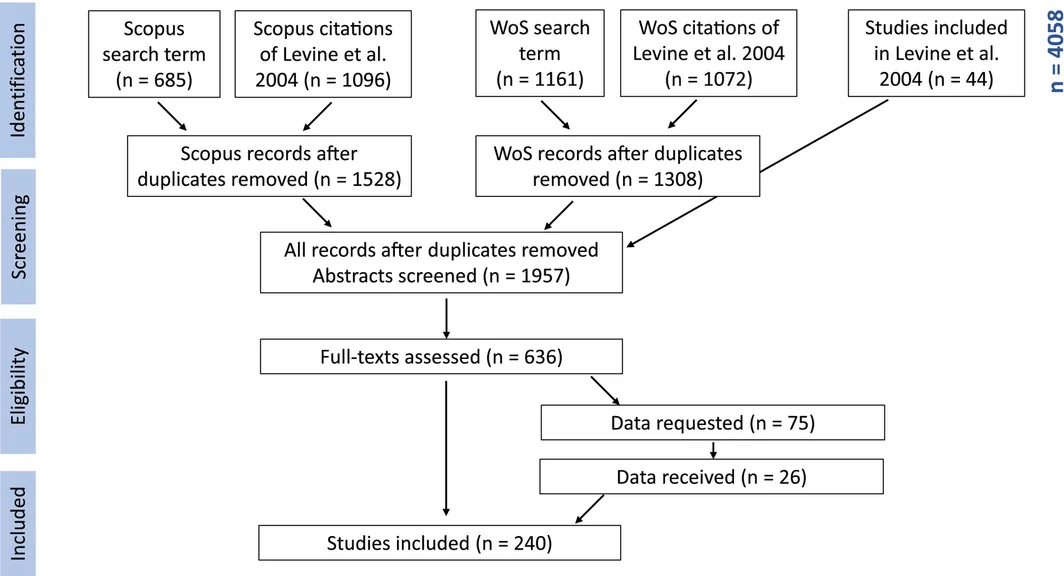
PRISMA (preferred reporting items for systematic reviews and meta‐analyses) flow diagram showing the process used to obtain the collection of studies included in the meta‐analysis. Of the 240 studies (with 1468 alien and 494 native observations; 62 studies included alien–native comparisons), 90 studied aboveground antagonism, 35 belowground antagonism, 116 competition, and 48 diversity–invasibility. Twenty‐eight studies investigated two biotic resistance mechanisms simultaneously.

To conduct our systematic review, we used CADIMA, an online open‐access tool to facilitate the systematic review process and enhance methodological consistency (Kohl *et al*., 2018). In the first step, we screened the title and abstract. We framed our research question and the associated inclusion criteria using the PECO approach (specifying population, exposure, comparator and outcome; Foo *et al*., 2021). For the title and abstract screening, a study was selected if it matched the following criteria: one or more alien plant species (‘population’); a biotic resistance treatment (‘exposure’) involving one or more biotic resistance mechanisms (aboveground antagonism such as consumption by herbivores or pathogens, belowground antagonism such as infection by soil pathogens, competition by native communities, and diversity–invasibility; for definitions see Table 1); a comparison of presence to absence of native communities, or a comparison of highest to lowest diversity levels (‘comparator’); a measure of performance or invasion success of the alien plant species (‘outcome’).

We considered experimental studies of varying types, including field, common garden, greenhouse, and laboratory experiments. All of them either manipulated the native community providing biotic resistance, or the alien plant(s), for instance by introducing the invader in different natural biotic resistance settings. We excluded purely observational or macroecological studies to avoid confounding environmental effects. We also excluded studies on biotic interactions that clearly focused on mutualists such as pollinators, seed dispersers, or mycorrhiza. We also did not consider weed–crop interactions and alien–alien interactions, that is, we only considered biotic resistance by native resident species or communities native to the regions the studies were conducted in. Our review and literature search did not focus on community assembly processes in general, thus we only included data on biotic resistance to native ‘invader’ species if they were studied along with alien species in the same experimental settings, to specifically test for differences in biotic resistance to alien *versus* native species.

In the second step, we checked the full text of the selected studies using additional criteria (performed solely by the first author, C.S.S., to maximise consistency). A study was selected if it had relevant extractable data on the performance or invasion success of alien plant species and if it was accessible (e.g. article in English or German, availability of full‐texts). For the relevant studies that lacked extractable data (or for which data were provided as indices such as competition index or log response ratios), we requested the data from the authors. We received the required data in a third of the requested cases (Fig. 1). Importantly, to avoid confounding biotic resistance effects with studies on the impacts of alien plants on native plants, those on competition or diversity–invasibility were only considered if the native species/community were established prior to the introduction of the alien species. We therefore excluded competition experiments in which the alien was sown/planted simultaneously with or before the native species. The study selection process returned 240 studies published between 1987 and 2023 (Fig. 1; see list of studies in online Supporting Information, Appendix S1).

From each selected study, we extracted qualitative and quantitative data on the characteristics of the study system, biotic resistance mechanisms and performance of alien species (and native species if given). The selected performance measures included vegetative performance, survival, reproductive performance, and population growth (for definitions see Table 1). For most of these measures, we extracted the mean performance, standard deviations (converting standard errors and confidence intervals if necessary), sample sizes of alien species under the biotic resistance treatment and the mean performance in the control treatment. For survival data (binomial data), we extracted the survivor counts and total counts (e.g. total number of established seedlings out of a total number of sown seeds). For competition, above‐ and belowground antagonism, we compared the presence of a native species/community *versus* its absence (of at least the dominant species/groups). For diversity–invasibility, we compared the highest richness level (usually species richness, occasionally functional group richness) to the lowest diversity level. For studies that considered two biotic resistance mechanisms, we also extracted data for the interaction treatment (e.g. alien performance under both aboveground antagonism and competition). No study that fulfilled our criteria considered three or more biotic resistance mechanisms. Note that for combinations of competition and diversity–invasibility, the interaction cannot be estimated, as diversity is zero in the absence of competition.

We chose one performance measure per category if available (i.e. one each for vegetative performance, survival, reproductive performance, or population growth). For this purpose, we followed a predetermined ranking of preference within each category, choosing population‐level measures over individual‐level measures as we considered them to be more important for the overall invasion success of a species (Table 1; and for a detailed overview of the distribution of these measures in our included studies, see Fig. S1). Whenever possible, if a study included multiple alien species or multiple native communities, we extracted data for each alien species or each native community separately (Table S1). In a few cases (7 studies, 53 observations), we could only extract means over several alien species. If multiple abiotic conditions were reported, we chose the highest and lowest level in the gradient design. If multiple sowing densities of alien species were reported, we chose the highest density. If multiple measurements over time were reported, we chose the final measurement. To extract data from figures, we used the Web Plot Digitizer (https://automeris.io/).

### (2) Effect size calculation

We analysed data using R v4.4.0 (R Core Team, 2024). We used the standardised mean difference, namely Hedges' *g* effect size statistic (Hedges & Olkin, 1985), to measure biotic resistance magnitude and direction. For continuous data, Hedges' *g* was calculated directly, and for binary data, we first estimated log odds ratios, which we then converted to Hedges' *g*. Because log odds ratios cannot be estimated in the presence of zero event counts, we added 0.5 to all counts for a specific observation when at least one of the counts was zero (Weber *et al*., 2020). We used the R package ‘esc’ to calculate effect sizes (Lüdecke, 2019).

### (3) Meta‐analysis models testing effects of biotic resistance (addressing research question 1)

To analyse the data, we fit multilevel mixed‐effects meta‐analytic models using the restricted maximum likelihood method. To account for statistical non‐independence due to shared controls or treatments, we assumed a correlation of 0.5 between pairs of observations with shared data when calculating sampling variance–covariance matrices (Noble *et al*., 2017), which were then used in the models. Across all the models, we used three random effects: article, group, and observation, with a group representing different performance measures on the same species in the same article, and observation referring to each individual observation. In the baseline model, we included two categorical variables as fixed effects (i.e. moderators): the performance measure and the biotic resistance mechanism (Tables 1 and 2). To decide whether the interaction between the two moderators should be included in the model, we fit a model with and without an interaction term using the maximum likelihood method and performed a likelihood ratio test. According to the test, including an interaction term improved the model fit and was thus retained. Effects with absolute values of studentized deleted residuals >3 (Viechtbauer & Cheung, 2010) based on the baseline model were considered outliers and were excluded from final analyses (45 observations). Additionally, to account for possible phylogenetic non‐independence in the data, we ran phylogenetically controlled analyses on the species‐level data (i.e. excluding the few observations across multiple alien species where species‐specific effects could not be extracted). For that, we standardised species names against The Plant List using the ‘U.Taxonstand’ v.1.1.3 package (Zhang & Qian, 2023), constructed a phylogeny using the ‘V.PhyloMaker2’ v.0.1.0 package (Jin, 2024), and provided a phylogenetic correlation matrix as an additional random effect in the models.

**Table 2:** Moderators used in the meta‐analysis, with their description, their respective categories (for factors) or ranges and units of measurement (for continuous variables), number of studies and observations. ^1^ = See Table 1 for order of preference; ^2^ = see https://www.iucnredlist.org/resources/habitat‐classification‐scheme; ^3^ = see https://doi.org/10.5281/zenodo.10039630 (Seebens *et al.*, 2017, 2018); ^4^ = Note that the native observations are not included in the other rows.

Moderator	Description	Categories or ranges	No. studies (no. alien observations)
Biotic resistance mechanism	Interaction type for which biotic resistance was studied	Competition Aboveground antagonism Belowground antagonism Diversity–invasibility	116 (688) 90 (391) 35 (173) 48 (216)
Performance measure	Measure of performance or invasion success of focal alien species (if available, one for each category extracted^1^)	Vegetative performance Survival Reproductive performance Population growth	184 (7751) 91 (498) 39 (189) 8 (30)
Species origin	Origin of the species with regards to the locality of the study	Alien Native	240 (1468) 62 (494)^4^
Study type	Experimental method used in the study	Common garden experiment Field experiment Greenhouse experiment Laboratory experiment	25 (151) 144 (888) 63 (385) 11 (44)
Habitat type	Studied habitat type, adapted from IUCN classification^2^	Forest Shrubland Savanna Grassland Wetland Marine (Multiple/undefined)	41 (172) 19 (91) 5 (27) 123 (963) 28 (90) 15 (42) 11 (83)
Invasion status	Invasion status of the focal alien species as given in the study	Alien non‐invasive Alien invasive	84 (790) 166 (678)
Spatial scale	Size of individual pot, plot, mesocosm etc. in which biotic resistance was studied	Continuous variable, ranging from 0.0005 to 5200 m^2^ with a median of 0.13 m^2^ (and one outlier of 80000 m^2^ and 56 undefined)	
Temporal scale	Study duration	Continuous variable, ranging from 0.02 to 1408 weeks with a median of 28 weeks (78 undefined)	
Minimum residence time	Difference between publication year and year of first record of focal alien species in the study region, as given in the study, or extracted from the FirstRecord database^3^ if available and species and country matched	Positive integer, ranging between 6 and 523 years with a median of 140 years (year of first record between 1500 and 2009; 350 unknown)	
Time‐lag bias	Year of publication of the respective study	Positive integer ranging between year 1987–2023	
Publication bias	Square root of the inverse of the effective sample size	Positive continuous variable	

All models (reported in Table S2) were parameterised using the function ‘rma.mv’ in the ‘metafor’ R package v4.6.0 (Viechtbauer, 2010). Marginal effects for individual moderators were calculated using the R package ‘emmeans’ (Lenth, 2024), providing proportional weights in calculations (reported in Table S3). We quantified heterogeneity of effect sizes as *I* ^2^ and the percentage of variation explained as marginal and conditional *R* ^2^.

### (4) Publication bias and time‐lag bias

We included publication year as a moderator to test for time‐lag bias. Time‐lag bias refers to the tendency to find larger effect sizes in older studies than in more recent ones when the speed of publication depends on the significance or impressiveness of study results (Koricheva & Kulinskaya, 2019; Nakagawa *et al*., 2022). To assess for publication bias, we incorporated the square root of the inverse of the effective sample size, which is comparable to the inverse of sampling variance after removing uncertain elements, as a moderator (Nakagawa *et al*., 2022). This approach is a modification of Egger's regression, which accommodates high heterogeneity and non‐independence. Only continuous performance measures could be included in this analysis.

### (5) Combined effects of multiple biotic resistance mechanisms (addressing research question 2)

We used the R package ‘multiplestressR’ (Burgess & Murrell, 2021) to compute effect sizes for pairwise interactions between individual biotic resistance mechanisms and classify them into null (i.e. the combined effect is additive), synergistic (i.e. the combined effect is greater than the sum of individual effects), antagonistic (i.e. the combined effect is smaller than the sum of individual effects), and reversal (i.e. when combined and additive effects have opposite directions) under the additive null model (Gurevitch, Morrison & Hedges, 2000). Only studies with a full factorial design and continuous performance measures were used for these analyses (i.e. 22 studies, as six studies measuring only survival had to be removed; see Appendix S1).

### (6) Effects of residence time and other moderators (addressing research question 3)

To further investigate whether biotic resistance varies with residence time, experimental methods or study system, we ran additional models including various moderators: residence time, study type, habitat type, invasion status, and spatial or temporal scale of the study (Table 2; and see Fig. S1 for an overview of the distribution of the various categories of each moderator in our data). We only included one extra moderator per model to not overfit the model to the data. For residence time, we tested for linear and quadratic effects. Since residence time was rarely provided in the studies, we complemented this information from the FirstRecord database (Seebens *et al*., 2017, 2018; Table 2). We also additionally tested if the effect of residence time depended on biotic resistance mechanism, performance measure, or any of the other moderators.

### (7) Biotic resistance to alien *versus* native species (addressing research question 4)

To compare the biotic resistance to alien species with resistance to native species, we fit a separate multilevel meta‐regression model on the data from studies that considered both species groups. As previously, we included the performance measure and biotic resistance mechanism, as well as the biogeographical origin (alien *versus* native) as moderators (Table 2). We included interactions between origin and performance measure, as well as origin and biotic resistance mechanism, given that, based on the likelihood ratio test, a model with these interactions outperformed the additive model.

## III. META‐ANALYSIS: RESULTS

### (1) Do native species consistently provide biotic resistance to alien plant species, which biotic resistance mechanisms exert the strongest effects, and how do these effects vary depending on alien plant performance measures?

Across the 1468 observations on alien performance from 240 studies, we found a significant overall effect of biotic resistance by native communities (Hedges' *g* = −0.77, 95% confidence interval: −0.86, −0.67), meaning that alien plant species consistently performed worse in the presence of native species or highly diverse communities compared to the absence of particular native species or low‐diversity native communities. Heterogeneity among effect sizes was high, with a total *I* ^2^ = 97.5% (among studies: 38.0%; among groups, i.e. multiple observations on the same species and same biotic resistance mechanism in the same study: 18.3%; among observations: 41.2%). Performance measure and biotic resistance mechanism explained 19.8% of the variation in the data (marginal *R* ^2^; conditional *R* ^2^ of 66.1%; Table 3).

**Table 3:** Meta‐analysis models, shown with their significance tests (*F* tests for factors, standardised effect sizes *t* for continuous variables; significant values (*P* < 0.05) are highlighted in bold font, marginally significant values (*P* < 0.1) in italics), amount of explained variation (*R* ^2^; marginal and conditional), heterogeneity between effect sizes (*I* ^2^; total and between study, group, observation, and where applicable, phylogenetic correlation) and number of observations (*n*). Note that each model included the interaction of biotic resistance (BR) mechanism and performance (perf.) measure. The residence time model additionally considered main effects of (linear and quadratic) minimum residence time (MRT). The models with other moderators included the main effect of one single extra moderator, except for the model testing for time‐lag and publication bias, which included both. The alien *versus* native model additionally included data on native species and tested for interactions with species origin. For the full Table including significance tests of all main effects included in the models, see Table S2. ^1^ = Given the marginal significance, results of the model without interaction are reported in Fig. S2. ^2^ = Factor with 6 levels: forest, shrubland, savanna, grassland, wetland, marine; ^3^ = Factor with 2 levels: terrestrial *vs*. aquatic; ^4^ = Factor with 2 levels: non‐invasive alien *vs*. invasive.

Research question	Model	Fixed effects	Significance	*R* ^2^ marginal (conditional) (%)	*I* ^2^ total (study, group, observation, phylogeny) (%)	*n*
1	Main model	BR mechanism × perf. measure	***F***_**6,1422**_ **= 2.6, *P* = 0.016**	19.8 (66.1)	97.5 (38.0, 18.3, 41.2)	1423
	Phylogenetic model	BR mechanism × perf. measure	*F*_*6,1349*_ *= 2*.*1, P = 0*.*053* ^*1*^	19.0 (67.9)	97.6 (34.4, 16.4, 38.6, 8.1)	1350
	Models to test for time‐lag and publication bias	Time‐lag bias Publication bias	*t = 1*.*936, P = 0*.*053* *t* = −1.032, *P* = 0.302	21.9 (74.4)	78.8 (29.9, 23.1, 25.9)	932
3	Residence time model	MRT Quadratic MRT	*t* = 1.447, *P* = 0.148 *t = −1*.*953, P = 0*.*051*	23.4 (69.5)	97.6 (37.8, 21.0, 38.8)	1079
	Models with other moderators	Study type Habitat type narrow^2^ Habitat type broad^3^ Log spatial scale Log temporal scale Invasion status^4^	*F*_3,1422_ = 1.9, *P* = 0.122 *F*_5,1343_ = 0.6, *P* = 0.699 *F*_1,1416_ = 1.8, *P* = 0.180 *t* = −0.144, *P* = 0.885 ***t* = 3.541, *P* < 0.001** ***F***_**1,1422**_ **= 4.0, *P* = 0.045**	21.1 (66.6) 21.6 (66.2) 20.0 (65.5) 19.5 (68.9) 20.9 (63.6) 20.5 (66.1)	97.5 (37.8, 18.5, 41.3) 97.4 (36.6, 18.8, 42.0) 97.5 (37.0, 18.4, 42.1) 97.5 (38.3, 21.6, 37.6) 96.5 (30.6, 21.5, 44.4) 97.5 (37.5, 18.4, 41.5)	1423 1344 1417 1369 1345 1423
4	Alien *vs*. native model	BR mechanism × perf. measure BR mechanism × origin Perf. measure × origin	***F***_**4,912**_ **= 2.6, *P* = 0.035** *F*_3,912_ = 1.5, *P* = 0.225 *F*_2,912_ = 1.7, *P* = 0.180	22.7 (62.1)	95.1 (36.5, 12.0, 46.6)	913
	Alien *vs*. native phylogenetic model	BR mechanism × perf. measure BR mechanism × origin Perf. measure × origin	***F***_**6,840**_ **= 4.5, *P* = 0.004** *F*_3,840_ = 1.0, *P* = 0.375 *F*_2,840_ = 1.4, *P* = 0.246	23.3 (62.8)	94.7 (39.1, 9.7, 45.9, 0.0)	841

The (non‐phylogenetic) main model with an interaction between performance measure and biotic resistance mechanism was significantly better compared to one with only additive effects (likelihood ratio test, *X* ^2^ (6 *df*) = 15.8, *P* = 0.015), indicating that the extent of biotic resistance the various mechanisms exerted varied with performance measure (Table 3, Fig. 2). When controlling for phylogenetic non‐independence, the model with the interaction term was however only marginally significantly better (*X* ^*2*^ (6 *df*) = 12.4, *P* = 0.053) compared to the additive one. Generally, competition exerted the strongest effect of biotic resistance, followed by aboveground antagonism. Diversity–invasibility also exerted biotic resistance (except for survival, where communities of high and low diversity did not differ). For the two performance measures for which estimates were available (i.e. vegetative performance and survival), the effects of belowground antagonism did not significantly differ from zero (Fig. 2). Due to a lack of studies, the effect on population growth could only be estimated for competition, where it was strongly negative, and for aboveground antagonism, where it was non‐significant and highly uncertain (large 95% confidence intervals). Pairwise differences between performance measures were only significant for competition, with population growth being most strongly and survival most weakly affected by competition (Fig. 2). Results of the phylogenetic interaction model are qualitatively similar to the main model (Table 3; S2 and S3), with a somewhat more negative overall adjusted mean Hedges' *g* of −0.86 and a wider 95% confidence interval ranging between −1.22 and −0.49 (total *I* ^2^ = 97.6%; among studies: 34.4%; among groups: 16.4%; among observations: 38.6%; phylogenetic relationships: 8.1%; marginal *R* ^2^ = 19.0%, conditional *R* ^2^ = 67.9%; Table 3). Results from a phylogenetic model with main effects only showed highly significant effects of biotic resistance mechanism (*F*_3,1343_ = 28.1, *P* < 0.001) and performance measure (*F*_3,1343_ = 7.7, *P* < 0.001) (see Fig. S2).

**Fig. 2:**
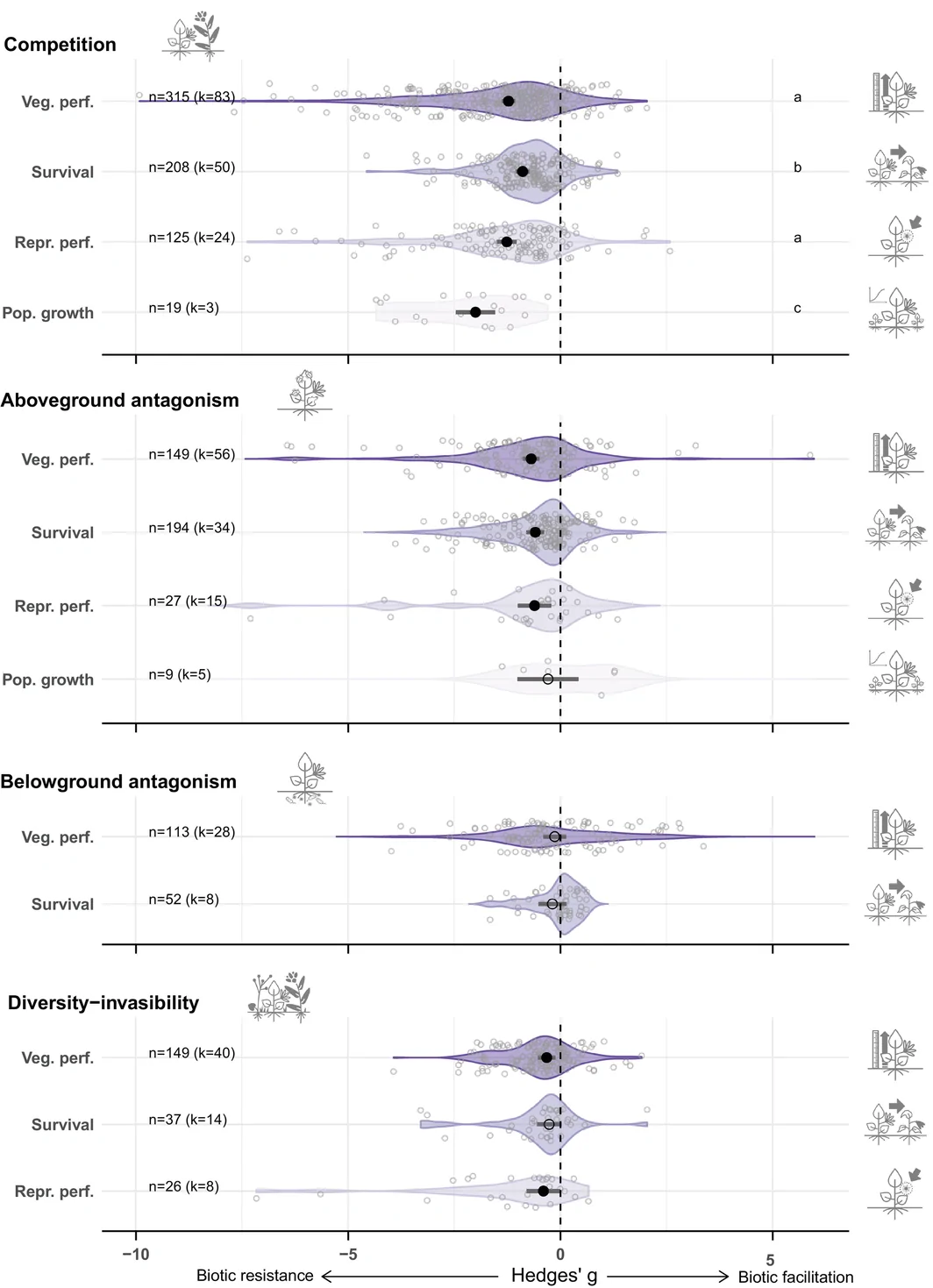
The magnitude and direction of biotic resistance (measured as Hedges' *g*) to alien plant invasions depending on the biotic resistance mechanism and plant performance measure. Negative values indicate biotic resistance (i.e. lower performance in the presence/high diversity of native species). For three biotic resistance × performance measure combinations, there were no data. The plot shows predicted effect sizes (black filled circles for significant effects, black unfilled circles for non‐significant effects) with 95% confidence intervals (dark grey lines) and the observed effect sizes (grey unfilled circles) and their distribution (in colour, with different colours for different performance measures). Different letters on the right indicate significant differences between performance measures within a biotic resistance mechanism (only indicated if any pairwise differences were significant). Sample sizes are given on the left as the number of observations (*n*) with the number of studies (*k*) in parentheses. Veg. perf. = vegetative performance; Repr. perf. = reproductive performance; Pop. growth = population growth. For better visibility, three outliers (two data points for aboveground antagonism/population growth at Hedges' *g* = −33.03 and *g* = 13.97, as well as one data point for belowground antagonism/vegetative performance at *g* = 14.94) were not plotted.

We did not detect any publication bias: when applying the Nakagawa *et al*. (2022) extension of Egger's regression, the square root of the inverse of the effective sample size did not have a significant effect. The year of publication, however, had a marginally significant effect, indicating a trend towards time‐lag bias (Table 3).

### (2) Do multiple biotic resistance mechanisms have additive effects, or do they interact, resulting in synergistic, antagonistic, or reversal effects on alien plants?

The combination of two biotic resistance mechanisms predominantly acted additively (43 of 65 observations), whereas synergistic (3), antagonistic (11), and reversal (8) combined effects were uncommon (Table S4). Consequently, we did not detect an overall synergistic effect (estimated effect size: 0.11, 95% confidence interval: −0.87, 1.09; Fig. 3). However, the sample size was small, given that few studies investigated more than one mechanism; additionally, we had to exclude observations on survival because the method we applied does not work for odds ratios.

**Fig. 3:**
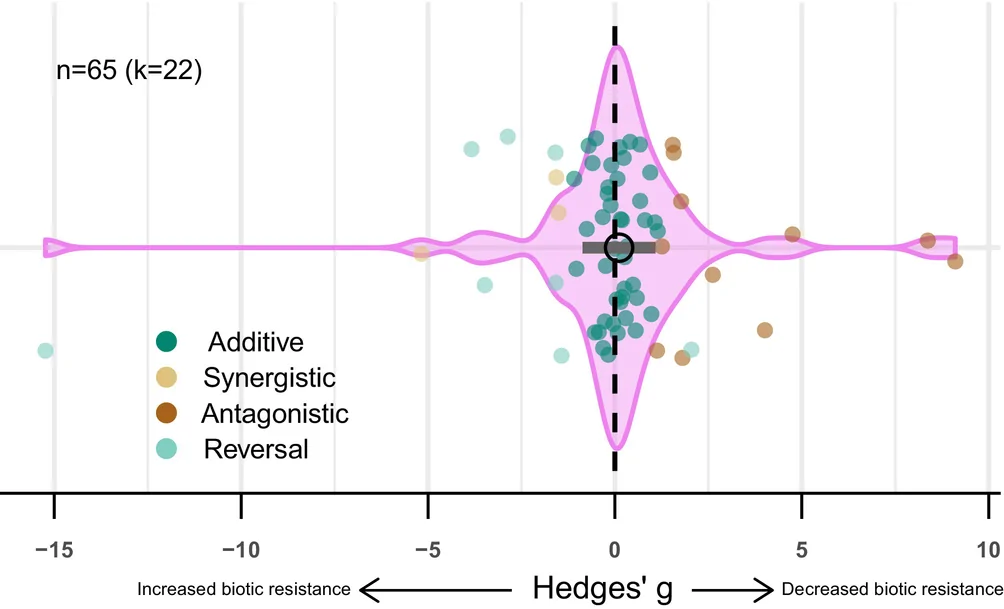
The magnitude and direction of pairwise interactions between mechanisms of biotic resistance to alien plant invasions (measured as Hedges' g), where an effect size of zero indicates additive effects (null expectation), negative values indicate a synergistic effect (i.e. more than the additive effect of the two biotic resistance mechanisms) and positive values an antagonistic effect. Reversal effects can be positive or negative. Thirteen studies (34 observations) are from combinations of aboveground antagonism × competition, 3 (20) from aboveground antagonism × diversity–invasibility, 2 (3) from aboveground × belowground antagonism, 1 (1) from belowground antagonism × competition and 3 (7) from belowground antagonism × diversity–invasibility. The plot shows the predicted effect size (black open circle) with 95% confidence interval (dark grey line) and the observed effect sizes (in colour; scattered along the arbitrary y‐axis for better visibility) and their distribution (in pink). Sample size is given on the left as the number of observations (*n*) with the number of studies (*k*) in parentheses. See Table S4 for details on which combinations of biotic resistance mechanisms resulted in which types of effects.

### (3) Does the strength of biotic resistance vary with the residence time of the alien species, the experimental methods, or the study system?

The study species ranged in regional minimum residence time from 6 to 523 years (with a mean of 146 years). A model including linear and quadratic effects of residence time was significantly better than one including only linear effects (likelihood ratio test, *X* ^2^ (1 *df*) = 3.9, *P* = 0.048). The significant quadratic relationship between residence time and the strength of biotic resistance (Hedges' *g*) indicated that biotic resistance decreased initially and then increased again after a residence time of approximately 200 years (Fig. 4).

**Fig. 4:**
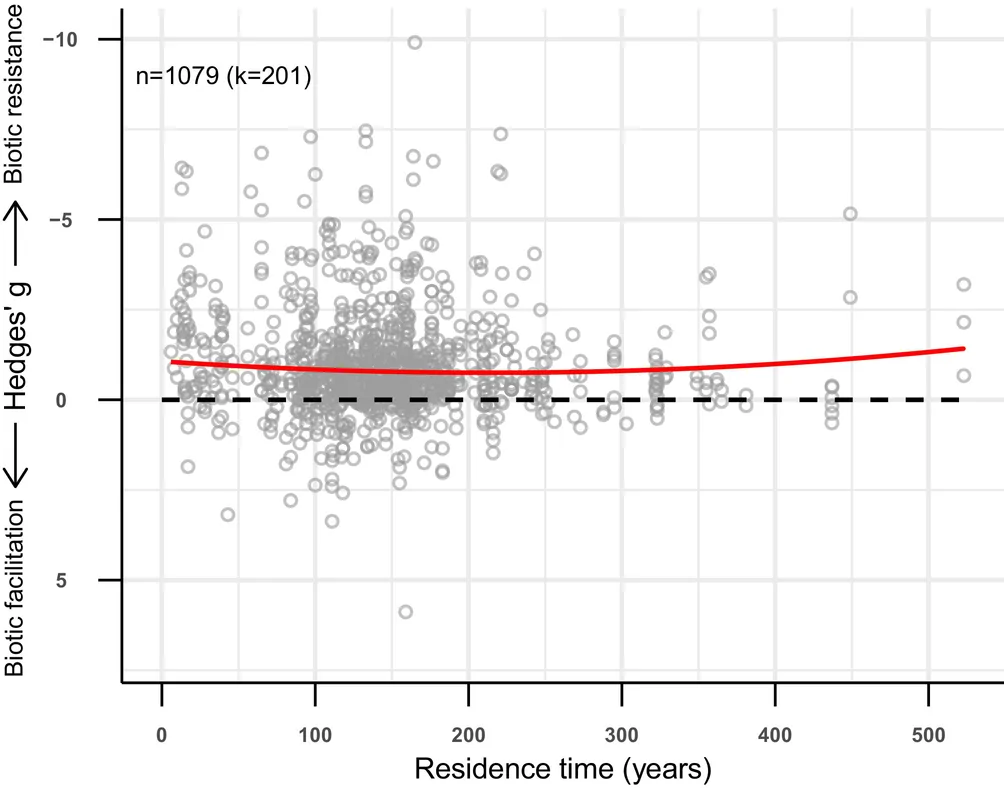
Predicted effect of residence time of alien plant species on the magnitude of biotic resistance (Hedges' *g*) to plant invasions, based on the meta‐analysis model including biotic resistance mechanism, performance measure and their interaction, as well as linear and quadratic effects of residence time. Negative values indicate biotic resistance (i.e. lower performance in the presence/high diversity of native species). Note that the y‐axis is plotted on an inverted scale and that for better visibility, an outlier (one data point at residence time = 100 years and Hedges' *g* = 14.9) was not plotted. Sample size is given on the left as the number of observations (*n*) with the number of studies (*k*) in parentheses.

We found only a few significant effects of experimental methods and study systems (Table 3). The magnitude of biotic resistance did not depend on the study type (field, common garden, greenhouse, or laboratory experiment), nor on the investigated habitat type, whether we compared differences among the six categories or more broadly between terrestrial and aquatic habitats (including wetland i.e. freshwater habitats, marshes, floodplains, and marine habitats).

While the log‐transformed spatial scale of the study did not influence biotic resistance, the log‐transformed temporal scale did, with decreased biotic resistance in studies of longer duration (Table 3). Counterintuitively, biotic resistance had a stronger, though marginally significant, effect on invasive compared to non‐invasive alien species (including alien species with undefined status, a few casual aliens, and established aliens).

When testing whether the effect of residence time varied with other moderators, the interactions between (linear and quadratic) effects of residence time and the other moderators were never significant (Table S5), with the exception of habitat type (although given the large number of included parameters, results should be treated with caution; see Fig. S3).

### (4) Does biotic resistance affect native plant species differently than alien invaders?

With the limited data available (i.e. 494 native and 546 alien observations from the subset of 62 studies that investigated species of both biogeographic origins), we detected no significant difference in biotic resistance to alien *versus* native species (Table 3). Although a model with two‐way interactions was better than an additive model, we found no significant interaction between biotic resistance mechanism and species origin. This suggests that native and alien species were affected in similar ways by the mechanisms of biotic resistance considered. Results from a phylogenetically controlled analysis were qualitatively similar (Table 3).

## IV. A BROADER PERSPECTIVE ON THE DYNAMICS OF BIOTIC RESISTANCE

Theory suggests that biotic resistance may change through time as both native resident species and invaders adapt, but long‐term studies are rare (Agrawal & Maron, 2022). We argue that the field is currently missing a clear conceptual overview on which to base predictions and further work. Here, we first develop a conceptual causal network graph exploring how biotic resistance may trigger and respond to eco‐evolutionary dynamics in invaded communities. We then provide a mathematical implementation of this causal network that illustrates how eco‐evolutionary dynamics cause biotic resistance to change over time.

### (1) Causal processes underlying temporal changes in biotic resistance

To illustrate the processes potentially inducing dynamics of biotic resistance, we constructed a conceptual causal network graph (Fig. 5), which is a tool for representing conceptual ideas about hypothesized or observed causal relationships (Heger, 2022). This graph depicts hypothetical temporal dynamics influencing biotic resistance, namely the impact of native communities on alien invaders. Here, we propose that the extent of biotic resistance depends on the invasiveness of the alien invaders (see purple boxes in Fig. 5, and references in Table 4), the invasibility of the native community (green boxes in Fig. 5, Table 4) and their interaction with the abiotic environment. Local establishment of new alien species can be influenced by propagule pressure (i.e. the number of introduced individuals of an invader) and colonization pressure (i.e. the number of introduced species). Higher propagule and colonization pressures increase the likelihood of establishment success, shaping the species or genotypic composition and density of alien invaders (links A–B in Fig. 5 and Table 4 for references). Eco‐evolutionary processes can act on both species (interspecific selection i.e. species sorting) and genotypes (intraspecific selection i.e. microevolution), leading to shifts in native and alien species/genotypic community composition and overall density, and as a result, changes in the invasiveness of the alien species and the invasibility of the native community over time (links C–H, in Fig. 5 and in Table 4). These selection pressures on composition and density, in turn, depend on species interactions and can be further influenced by changes in abiotic factors (links I–J, in Fig. 5 and in Table 4). Additionally, temporal changes in the invasibility of the native community can also in turn influence the local establishment of new alien species or genotypes (link K in Fig. 5 and in Table 4).

**Fig. 5:**
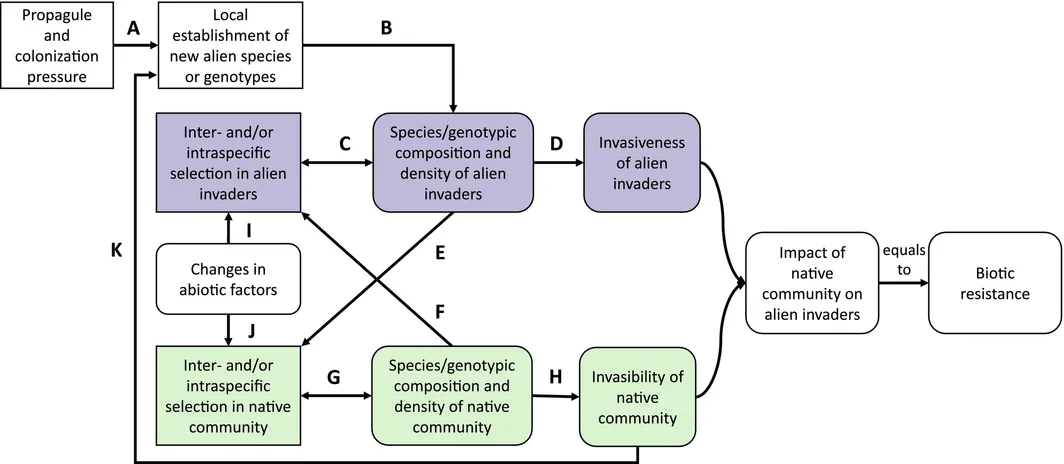
Conceptual causal network graph showing hypothetical temporal dynamics of biotic resistance. The graph shows how changes in the native community (green boxes), the alien invaders (purple boxes) and the abiotic environment may lead to changes in invasiveness and invasibility, and thus in biotic resistance (or possibly, biotic facilitation). Square boxes indicate processes, rounded boxes represent states, and arrows indicate key causal links resulting in a change in either a state or a process. Feedback loops lead to a complex interplay between ecological and evolutionary changes in the native community and in the alien species. For every causal relationship represented in the graph (bold capital letters), examples from the literature are given in Table 4.

**Table 4:** Examples of studies supporting mechanistic links in the causal network graph illustrated in Fig. 5. Note that only the most relevant aspects of the studies (e.g. not all performance measures) are represented.

Causal link	Study species/system	Mechanism(s) involved	Performance measures	Residence time of an alien plant (if considered)	Key finding(s)	Reference
A	48 alien and 45 native plant species sown in grasslands in Europe	Neutral processes, competition, aboveground antagonism	Survival (establishment success)		Establishment success of the alien species promoted by propagule pressure, but also by tolerance to herbivory and competition	Kempel *et al*. (2013)
B	Five alien and five native species in Europe grown in soil conditioned by alien and native heterospecifics	Belowground antagonism	Vegetative performance (aboveground biomass)		Heterospecific soil conditioning by alien plants facilitated success (competitive ability) of later arriving aliens compared to natives, due to modification of soil microbiome	Zhang *et al*. (2020)
C	*Mikania micrantha*, alien in China	Competition	Vegetative performance (aboveground biomass, competitive ability)	>30 years (using spread distance from source)	Increased competitive ability in older *M*. *micrantha* populations	Huang & Peng (2016)
C, D	*Alliaria petiolata*, alien in the USA	Competition	Population growth (change in cover), phytotoxin concentration	>50 years	Intraspecific interactions select for lower phytotoxin production in *A*. *petiolata*, resulting in lower invasiveness in older populations	Lankau *et al*. (2009)
E	Response of native butterfly *Euphydryas editha* to its native host plant *Collinsia parviflora* and alien host plant *Plantago lanceolata* in the USA	Aboveground antagonism	Oviposition preference of the butterfly	>20 years	Oviposition preference of the native butterfly switched from the native to the alien host	Singer & Parmesan (2018)
E	Response of native plant *Vulpia microstachys* to *Bromus hordeaceus*, alien in the USA	Competition	Population growth (lambda), vegetative performance (competitive ability of the native plant)	(Sympatric *vs*. allopatric sites)	Evolution of the native *V*. *microstachys* to better compete for resources used by *B*. *hordeaceus*	Germain, Srivastava & Angert (2020)
F	*Senecio inaequidens*, alien in Europe	Competition	Reproductive (no. flowerheads) and vegetative (aboveground biomass) performance	Up to 111 years	Genetic differentiation of *S*. *inaequidens* according to the intensity of competition with native species	Lachmuth, Durka & Schurr (2011)
F	*Impatiens glandulifera*, alien in Europe	Aboveground antagonism	Leaf damage, concentration of defence compounds	Up to 85 years	Greater herbivore resistance in *I*. *glandulifera* due to increased attack rates from native herbivores over time	Gruntman *et al*. (2017)
G	*Oenothera biennis*, native in the USA	Aboveground antagonism, competition	Vegetative performance (competitive ability), concentration of defence compounds, survival (seed predation)		Reduced herbivory selected for increased competitive ability but reduced herbivore resistance in *O*. *biennis*	Agrawal *et al*. (2012)
H, K	Alien tree species recorded in forest plots globally	Diversity–invasibility	Vegetative performance (abundance of alien tree species)		Greater native forest diversity was associated with lower severity of alien tree invasions	Delavaux *et al*. (2023)
H, K	Spontaneous native and alien colonizers of experimental plots of *Solidago altissima*, native in the USA	Diversity–invasibility	Vegetative performance (aboveground biomass of spontaneous colonizers)		Intraspecific diversity (no. of *S*. *altissima* genotypes) reduced the biomass of spontaneous colonizers	Crutsinger, Souza & Sanders (2008)
I	*Lythrum salicaria*, alien in the USA	Adaptation to novel climatic conditions	Reproductive performance (no. fruits)	Up to 100 years	Fast adaptive differentiation of *L*. *salicaria* along a climatic gradient	Colautti & Barrett (2013)
J	*Schoenoplectus americanus*, native in the USA	Adaptation to increased nitrogen levels	Vegetative performance (belowground biomass), ecosystem functioning	*ca*. 50 years (descendant and ancestral genotypes)	Evolutionary differentiation in belowground vegetative traits of *S*. *americanus* and its predicted consequences for ecosystem functioning	Vahsen *et al*. (2023)

Previous studies have addressed the various processes related to biotic resistance illustrated in Fig. 5. We highlight examples of studies for individual causal relationships in Table 4. We stress that this framework is intended to be generally applicable to any biotic resistance mechanism or performance measure; Table 4 is intended solely to show the plausibility of the mechanistic links and should not be interpreted as evidence that any one link is more likely to be seen in a given system or with a specific type of biotic resistance. In the following subsection, we build on this conceptual framework by introducing a simple mathematical model that captures these various determinants of temporal dynamics of biotic resistance. Most of the studies in the meta‐analysis were limited to a single invader (Table S1), but most real‐world ecological scenarios can have multiple introduced species in an area, so our conceptual and mathematical models are broadened to this scope (considering multiple alien species).

### (2) Disentangling the determinants of temporal dynamics of biotic resistance

We highlight three key aspects that are typically absent from studies of biotic resistance (Fig. 5): species sorting in both natives and invaders (facilitated by interspecific variation); within‐species selection in natives and invaders (facilitated by intraspecific variation); and changes in the density of natives. Instead, studies typically focus on invasion into stationary communities, but do not assess how biotic resistance is altered by eco‐evolutionary changes in natives and invaders.

The model we present below focuses on steps B–K in Fig. 5 and analyses how density dynamics of invader and native species (Fig. 6) may result from the selection response of the community and potential invaders. These responses largely depend on pre‐existing variation in invasibility and invasiveness (Fig. 7) and ultimately impact biotic resistance over time (Fig. 8). The overall variation in invasibility of the native community (*V*_*N*_) has an interspecific component (i.e. differences among resident species in how strongly they contribute to invasibility; *V*_*N*,interspecific_) and an intraspecific component (i.e. the average variation within resident species; *V*_*N*,intraspecific_). Hence, the invasibility of the community changes over time due to species sorting (e.g. increasing frequency of more competitive species) and simultaneous microevolutionary changes within species (Vellend, 2017; link G in Fig. 5). Species sorting is likely to result in stronger changes in invasibility since trait variation is typically greater among species than within species (e.g. Siefert *et al*., 2015). Hence, initially more diverse communities should develop biotic resistance more rapidly (Fig. 7), as posited by the diversity–invasibility hypothesis (Elton, 1958). Similarly, the invasiveness of the invaders can change over time (link C in Fig. 5) if there is variation in invasiveness (*V*_*I*_), which also depends on both interspecific variation in the pool of potential invaders (*V*_*I*,interspecific_) and on intraspecific variation within individual invader species (*V*_*I*,intraspecific_). Interspecific variation in invasiveness should increase with colonization pressure, and intraspecific variation in invasiveness should increase with propagule pressure (link B in Fig. 5).

**Fig. 6:**
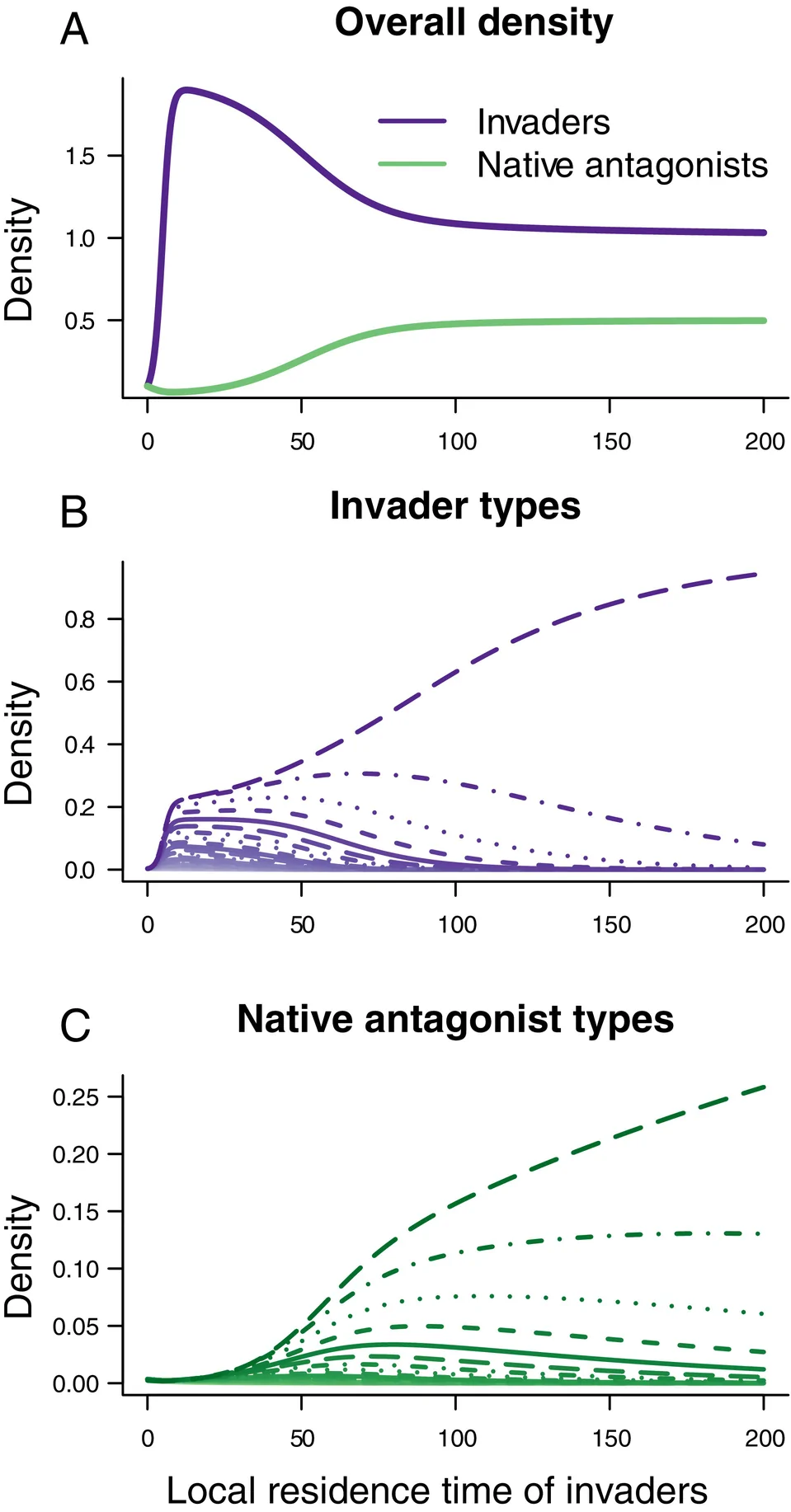
An example of invader–native antagonist dynamics described by the model. (A) Dynamics of the overall density of invaders (purple) and native antagonists (green). (B) Dynamics of invader types (species or genotypes, represented as different line types) that differ in intrinsic population growth rate *r*. (C) Dynamics of native antagonist types (species or genotypes, represented as different line types) that differ in the rate *a* at which they attack invaders. Note that native antagonists are initially rare but then increase as antagonist types with increasing attack rates on the invaders are selected for. Note that residence time and density have arbitrary units.

**Fig. 7:**
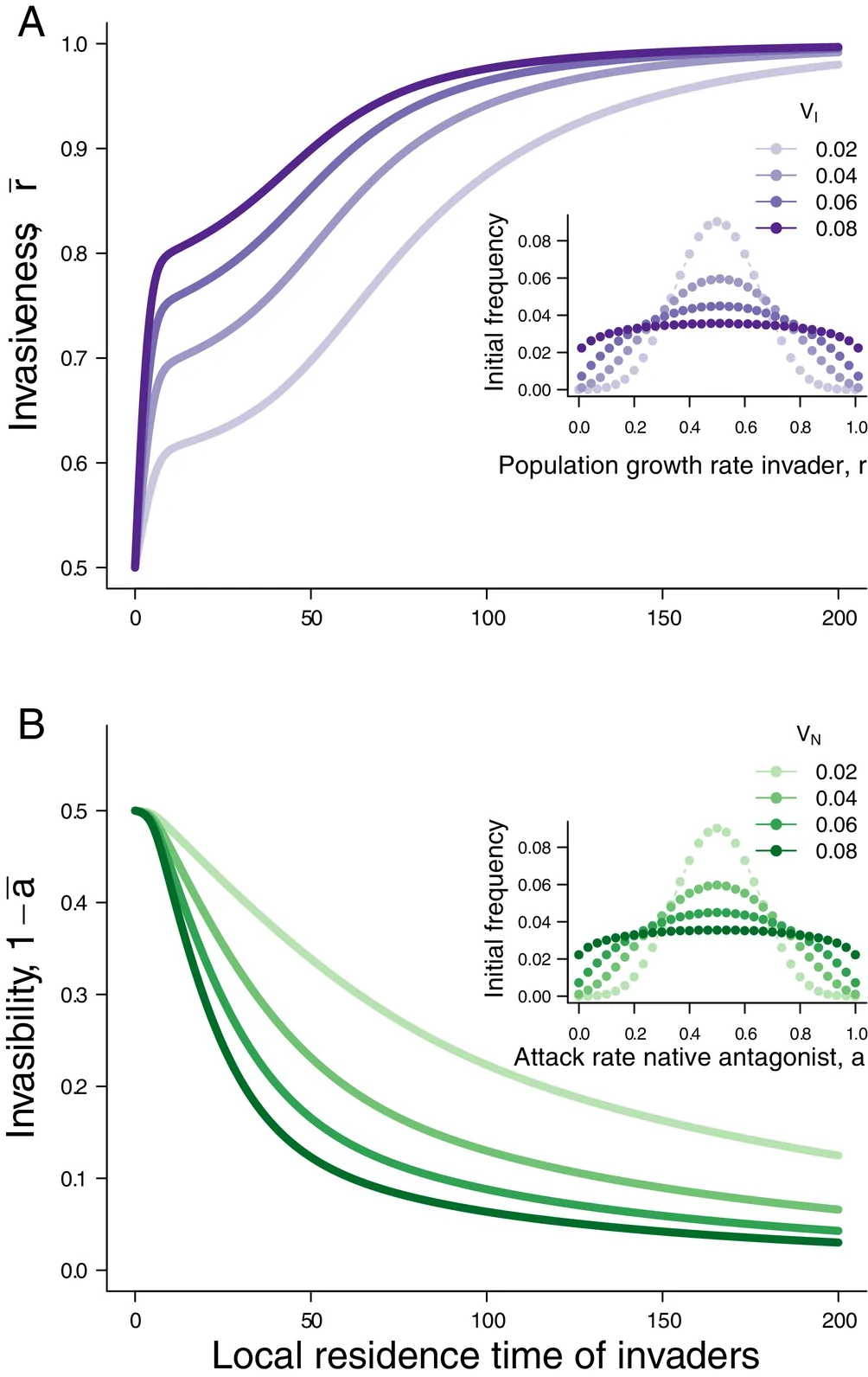
(A) The initial variance *V*_*I*_ of the intrinsic population growth rate of invaders *r* determines the speed at which invasiveness ($\bar{r}$) increases with local residence time, thereby reducing biotic resistance. See the insert for alternative initial distributions of *r* that have the same mean but differ in *V*_*I*_. (B) Similarly, the initial variance *V*_*N*_ of the attack rate of native antagonists *a* determines the speed at which community invasibility $(1-\bar{a}$) decreases with local residence time, thereby promoting biotic resistance. See the insert for alternative initial distributions of *a* with the same mean but different *V*_*N*_.

**Fig. 8:**
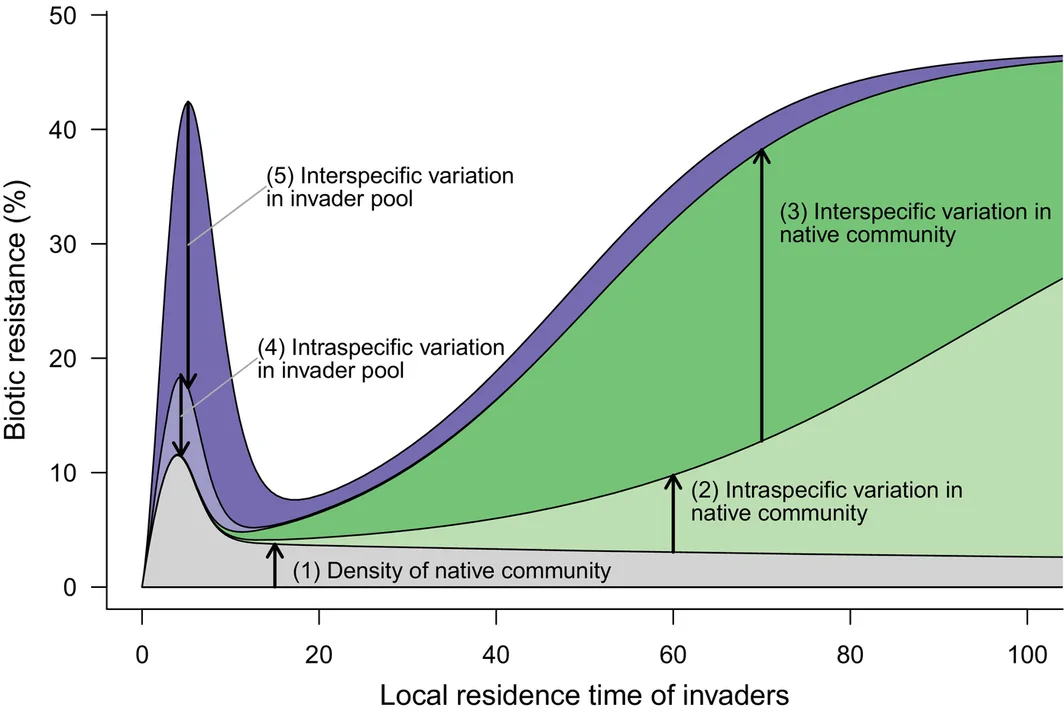
Biotic resistance and its determinants change with local residence time. The upper boundary of each coloured area represents biotic resistance in a given model scenario. Starting from the horizontal axis (representing a scenario in which native antagonists are absent and invaders have full inter‐ and intraspecific variation), we added (1) native antagonists without variation, (2) intraspecific variation in antagonists, and (3) both inter‐ and intraspecific variation in antagonists. Subsequently, we lowered variation in invaders to represent (4) only interspecific variation (removing intraspecific variation in invaders), and (5) no variation (removing interspecific variation in invaders). The coloured areas thus represent changes in biotic resistance that are due to density effects of the native community (grey) as well as intra‐ and interspecific variation in the native community (light and dark green), and decreases in biotic resistance caused by intra‐ and interspecific variation in the invader pool (light and dark lilac). [Correction added on 24 September 2026, after first online publication: In the artwork for Figure 8, the text in labels (3) and (5) has been corrected from “Intraspecific” to “Interspecific”.]

Specifically, we modelled biotic resistance by a native community of antagonists *N* against invaders *I* with an eco‐evolutionary version of the Lotka–Volterra model for predator–prey interactions. Our goal was to provide a simple mathematical implementation of the conceptual causal network (Fig. 5). For this reason, we modelled antagonistic rather than competitive interactions between natives and invaders since a model representing competition would have to explicitly resolve resource dynamics. We also assumed that abiotic conditions are constant (so that links I and J in Fig. 5 do not alter biotic resistance). This model is sufficient to illustrate multiple determinants of biotic resistance and how their importance changes with residence time. Specifically, we used the discrete trait Lotka–Volterra model of Cortez & Weitz (2014). In this model, invaders consist of different types *i* that represent species and/or genotypes differing in intrinsic population growth rate, *r*_*i*_ (a key dimension of invasiveness). Similarly, the community of native antagonists comprises different species and/or genotypes *j* that differ in the rate *a*_*j*_ at which they attack invaders (a dominance of native antagonist types with low *a* means high invasibility).

The temporal dynamics of the density of invader type *i*, *I*_*i*_, are given by.

(1) $${I_{i}}^{\prime }=r_{i}\;I_{i}\;\left(1-I_{i}/K\right)–\sum_{j}I_{i}a_{j}N_{j},$$

and the density of native type *j*, *N*_*j*_, changes according to

(2) $${N_{j}}^{\prime }=c\;\sum_{i}I_{i}a_{j}N_{j}-{\mathrm{mN}}_{j},$$

where *K* is the carrying capacity of invaders, *c* is the conversion rate of consumed invader biomass into antagonist biomass, and *m* is the mortality rate of antagonists. For the following simulations, we considered 30 distinct types of both invaders and native antagonists. These different types have values of intrinsic population growth rate *r* and antagonist attack rate *a*, respectively, that are spaced equidistantly from 0 to 1 (for details see Appendix S2). The initial mean value of *r* and *a* was set to 0.5, representing limited pre‐adaptation of invaders to the local environment and limited co‐evolutionary experience of the antagonist. The initial variance in *r* and *a* is *V*_*I*_ and *V*_*N*_, respectively.

Model simulations illustrate several points about how invaders, native communities and biotic resistance change with local residence time of invaders. Overall invader density initially increases to close to carrying capacity before an increase in the overall density of native antagonists lowers invader density until the system reaches an equilibrium (Fig. 6A). These seemingly simple dynamics arise from more complex dynamics at the level of individual types of invaders and native antagonists (Fig. 6B,C). The dynamics of individual invader types are driven by selection for increased invasiveness (measured as the invaders' mean population growth rate $\bar{r}$) that cause particularly strong increases in invasiveness during the initial surge of invader density (Fig. 7A). The native community experience selection for decreased invasibility (measured as the complement of mean attack rate, 1 − $\bar{a}$) that result in a more gradual decline of its invasibility (Fig. 7B). Higher intra‐ and/or interspecific variance of invaders, *V*_*I*_, increase the speed at which their invasiveness increases (Fig. 7A). In contrast, higher intra‐ and/or interspecific variance of native antagonists, *V*_*N*_, increase the speed at which community invasibility declines (Fig. 7B).

The model can quantify how different determinants of biotic resistance change with residence time. To illustrate this, we compared invasion dynamics predicted for several alternative scenarios (Appendix S2; Table S7): starting with a scenario of maximum inter‐ and intraspecific variation in invaders but no native antagonists (and hence no biotic resistance), we sequentially considered native antagonists without variation (*V*_*N*_ = 0), with only intraspecific variation and with both inter‐ and intraspecific variation (assuming that 75% of overall variance resides among species, see Siefert *et al*., 2015). Subsequently, we lowered the variation in invaders to represent only interspecific variation or no variation (*V*_*I*_ = 0). For each scenario and timestep, we measured biotic resistance as the proportional reduction in invader density relative to maximum invasion success (for maximum invader variation and in the absence of native antagonists).

(3) $$\mathrm{BR}=\left[I_{\max}-I\right]/I_{\max}$$

where *I* is the overall invader density in the respective scenario and *I*_max_ is the invader density in the scenario of maximum invasion success (see Appendix S2, Table S7 for details).

For the scenarios shown in Fig. 8, biotic resistance initially increases due to the mere effect of native antagonist density, which increases in response to the increase in invaders. In this initial phase, biotic resistance is strongly reduced by selection for increased invasiveness enabled by intra‐ and interspecific variation among invaders (Fig. 8). Subsequently, biotic resistance drops since invader density approaches carrying capacity *K* in all scenarios. Thereafter, however, biotic resistance builds up again due to intra‐ and interspecific selection for decreased invasibility in the native community. This increase in biotic resistance is counteracted somewhat by selection for increased invasiveness in the invaders (Fig. 8). Thus, in the later stages of invasion, we predict that intra‐ and interspecific diversity of the native community is much more important than native density in resisting invasion, but empirical evidence supporting or refuting this is severely lacking. While the results shown in Fig. 8 are somewhat contingent on the precise parameterization chosen, they show (*i*) that biotic resistance changes with residence time, (*ii*) that it depends on density dynamics of the native community as well as on inter‐ and intraspecific variation in both the native community and the potential alien invaders, and (*iii*) that the importance of these dynamics for biotic resistance changes with residence time. The overall dynamic of biotic resistance decreasing then increasing again was also observed in the meta‐analysis (Fig. 4), despite our mathematical model being developed independently of the meta‐analysis results. In the meta‐analysis, temporal dynamics are treated indirectly by using residence time as a proxy (rather than actually being able to follow a given invader through time); here, our model provides a theoretical basis for how this pattern could be generated and underlines the need to study the temporal dynamics of biotic resistance.

## V. DISCUSSION

Here, we integrated a global synthesis of empirical studies, a conceptual causal network and a mathematical model to advance our understanding of biotic resistance to alien plant invasions and its temporal dynamics. Our meta‐analysis of 240 experimental studies showed that competition, aboveground antagonism and diversity of resident communities reduced invasion success, but the magnitude of this reduction depended on the plant performance measure (Fig. 2). From the limited number of studies combining two biotic resistance mechanisms, additive effects dominated, indicating that the mechanisms do not reinforce each other (Fig. 3). Few empirical studies to date have focused on temporal dynamics of biotic resistance, and important determinants of biotic resistance are hitherto poorly studied. However, across studies, we found that biotic resistance changed with the invader's residence time, first decreasing and then increasing (Fig. 4). Similar dynamics are predicted by the independently developed mathematical model focusing on antagonism (Fig. 8), although this model also reveals that dynamics of biotic resistance can be more complex. We argue that biotic resistance involves both aspects of species invasiveness (i.e. intrinsic attributes of an alien species such as competitive ability) and community invasibility (i.e. biotic and abiotic attributes of the recipient native community), which together determine the impact of a native community on the alien invader (Fig. 5) and may change over time. We stress that such changes in biotic resistance can be due to both interspecific selection (a change in biotic resistance due to changes in species composition and density) and/or intraspecific selection, that is, evolution in individual populations of alien and/or native species. Below, we discuss how biotic resistance has been studied so far and highlight important avenues for future research.

### (1) Empirical evidence of biotic resistance: findings from the meta‐analysis

Competition from native plants had the strongest effect on alien plant performance and was studied the most (Figs 2, S1 and S2). Many studies that examined biotic resistance *via* competition were field or common garden experiments that compared the effects of intact natural communities *versus* vegetation removal (e.g. by clipping, herbicide application, or ploughing; te Beest, Mpandza & Olff, 2015; Connolly, Powers & Mack, 2017) or artificially constructed native communities *versus* bare soil (Mason, French & Jolley, 2017; Brendel, Schurr & Sheppard, 2023). In these studies, alien species were usually added to the experimental units by sowing or planting, and only a few studies have used this setup to examine the effect of native plant communities on spontaneous colonization by alien plants from the surrounding vegetation (Heckman *et al*., 2017; Schuster *et al*., 2022). Some studies targeted specific competition mechanisms such as allelopathic effects from leaf leachates of native plants (Thiébaut, Tarayre & Rodríguez‐Pérez, 2019; Yuan & van Kleunen, 2022) or canopy shading by native woody vegetation (Becerra & Bustamante, 2011; Saccone *et al*., 2013).

Following competition, aboveground antagonism had the next strongest effect and was also frequently studied (Figs 2, S1 and S2). Most studies on aboveground antagonists as a biotic resistance mechanism were field experiments that examined the effect of herbivore or granivore exclusions (e.g. using fences or insecticides; Pearson *et al*., 2014; Wells *et al*., 2023). Other studies looked at the effects of herbivory by specific insect species under lab or greenhouse conditions (Calvo *et al*., 2019; Shan & Hou, 2023). However, only a handful of studies examined the combined effect of multiple natural enemies (e.g. both mammals and insects; Ashton & Lerdau, 2008; Korell *et al*., 2017), and even fewer examined the effect of aboveground pathogens (Parker & Gilbert, 2007). In contrast, studies on belowground antagonists, which were generally fewer, examined mostly the effect of soil microbiota and plant–soil feedback (using live *versus* sterilised soil; Andonian *et al*., 2012; Connolly, Carris & Mack, 2018), while only a single study examined the effect of native soil herbivores (Jin *et al*., 2022). Because plant–soil feedback experiments usually do not separate beneficial and antagonistic soil microbiota, we could not fully isolate the belowground antagonistic mechanism. This may also be a major reason for the apparent lack of significant effect of this mechanism (Figs 2 and S2). Recent advances in next‐generation sequencing could make it possible to better understand the role of soil antagonists as a biotic resistance mechanism (Dawson & Schrama, 2016; Yannelli *et al*., 2022).

Plant species diversity as a biotic resistance mechanism has been studied in the field or common gardens, with randomly assembled synthetic plant communities of varying species richness (Adomako *et al*., 2019; Hou & Wang, 2023). Our results confirm the importance of diversity in modulating biotic resistance of communities. In the studies analysed, niche complementarity of species and the resulting lack of empty niches for new invaders (Elton, 1958) was considered the main cause of biotic resistance. However, species diversity can also contribute to biotic resistance *via* the sampling effect, whereby more diverse communities are more likely to contain resistant competitors (Crawley *et al*., 1999). Some studies also considered functional diversity (Hooper & Dukes, 2010) or intraspecific diversity (Crutsinger, Souza & Sanders, 2008; Table 4) of plants. However, studies considering species diversity of antagonists such as herbivores or pathogens, which might be an important biotic resistance mechanism, are currently lacking.

Regarding other moderators besides residence time (discussed below), only temporal scale significantly affected biotic resistance, with longer studies reporting lower biotic resistance. Given that the longest duration was 1408 weeks (i.e. 27 years, with the median duration being 28 weeks), this is less likely to be a result of evolutionary changes. We also found a trend for biotic resistance against invasive plants to be stronger than resistance against non‐invasive alien plants. This somewhat surprising result could be due to adaptation of the native community, as invasive species likely reached larger densities than non‐invasives and hence the native species co‐occurring with invasives are more likely to have experienced selection pressures towards higher resistance against them (e.g. Germain *et al*., 2020; Table 4; Fig. 5 and see our mathematical model). However, it may also be an artefact due to different definitions of ‘invasive’ (see Soto *et al*., 2024), as the assignment to non‐invasive and invasive was done based on the information given in the original publications.

As we detail below, to better understand the effectiveness of biotic resistance in reducing invasions, future studies should co‐examine multiple biotic resistance mechanisms and their interactions, across different groups of competitors or antagonists, and on various performance measures (see Section V.2). Also, the effect of biotic resistance to alien invaders should be compared to colonization by native range expanders to tease apart species origin from species identity (see Section V.3). Most importantly, we need more studies incorporating a temporal perspective on biotic resistance to (see Section V.4).

### (2) Research gaps: how biotic resistance depends on (multiple) mechanisms and performance measures

Our meta‐analysis showed that the effect of the biotic resistance mechanism depended on the plant performance measure. Plant growth, survival, and reproduction are different aspects of a plant's life history, and the impacts of biotic resistance on these various performance measures may differentially influence the success of alien populations. For instance, a recent meta‐analysis showed that the extent of alien species' superior performance compared to natives varied depending on whether survival, growth, reproduction or other measures were considered (Sheppard & Lüpke, 2024). Still, in our meta‐analysis, 70% of the studies (166/240) looked at only a single performance measure, and only 5% of the studies (13/240) considered some measure of each of growth, survival and reproduction to fully capture demographic impacts (Ramula *et al*., 2008). The finding that biotic resistance by competition was generally less negative for survival compared to the other three performance measures (Fig. 2), could be explained with lower resource availability reducing growth and reproduction, while still allowing survival. Indeed, in many experiments, even the most suppressive communities fail to achieve complete exclusion of the alien species (e.g. Yannelli, MacLaren & Kollmann, 2020). Furthermore, when resident native plants provide a beneficial microclimate, this may even enhance survival. Studies on population growth were rare. The effect on population growth was strongly negative for competition but neutral for aboveground antagonism (note however the small number of studies). Weaker effects of biotic resistance by aboveground antagonism compared to competition could be due to enemy release (Keane & Crawley, 2002), where specialist native enemies avoid alien plants. For competition, where we found the highest number of studies for all performance categories, individual demographic variables were not as strongly affected as population growth (which is determined by growth, survival, and reproduction). This implies that snapshot measurements of plant performance are not necessarily good surrogates for population dynamics (Sheppard & Lüpke, 2024).

The interaction of multiple biotic resistance mechanisms and how they influence different plant performance measures has rarely been considered in studies of biotic resistance. Alien species arriving in a new region will always face some degree of competition, as well as aboveground and belowground antagonism simultaneously. Yet, in our review, only 12% of the studies (28/240) considered the possible interactions of multiple biotic resistance mechanisms. While we found no overall evidence for synergistic interactions between different mechanisms (Fig. 3), individual studies show the potential for these interactions to occur. For example, biotic resistance can be enhanced through synergistic interactions between competition and herbivory (e.g. Knapp, Fownes & Harrington, 2008; Silveira & Thomaz, 2023), though other studies reported no synergies between these variables (e.g. Erneberg, 1999; Pearson, Potter & Maron, 2012), suggesting certain conditions or species combinations are more effective than others at limiting invasion. The combination of competition and aboveground antagonism was studied the most, but with the limited sample size for other combinations no clear pattern can be detected regarding which combination of mechanisms may more likely result in additive, synergistic, antagonistic, or reversal effects (Table S4), although this would be an important research avenue. Indeed, biotic resistance has also been proposed as an important tool for ecological restoration (Funk *et al*., 2008; Byun, de Blois & Brisson, 2018). Without more studies simultaneously testing multiple biotic resistance mechanisms, we cannot generalise about optimal ways to strengthen natural biotic controls against invasion.

While examining both multiple biotic resistance mechanisms and multiple performance measures is ideal, only 3% of the studies (7/240) did so. Two studies in our review highlight the importance of this approach. Studying the invasive *Cirsium vulgare*, Suwa, Louda & Russell (2010) found additive effects of competition and aboveground herbivory (and only a weak main effect of competition) on growth and reproduction. However, Suwa & Louda (2012) found strong and synergistic effects of competition and aboveground herbivory on *C*. *vulgare*'s seedling survival. Therefore, evidence for biotic resistance is conditional on the type(s) of mechanism studied and the plant responses measured. To fully understand the nuances of how biotic resistance may affect alien species, future studies should not only consider the interactions of multiple biotic resistance mechanisms but their effects on multiple performance measures as well.

### (3) Research gaps: biotic resistance on alien *versus* native species

The biotic resistance mechanisms we discuss, that is, competition, above‐ and belowground antagonism, and diversity–invasibility, also apply to the colonization of a community by non‐resident native species. With the limited dataset, we could not detect differences between biotic resistance to alien *versus* native species. We note that here we certainly did not capture a complete set of studies on colonizing native species, as we only included studies on natives if they were incorporated in the same experiment as aliens. Thus, with more studies, one may be able to differentiate in what aspects biotic resistance to aliens differs from general community assembly processes. For instance, initial biotic resistance upon the first arrival of an alien species is expected to differ from natives due to an introduction bias in alien species (e.g. a human preference for introducing fast‐growing, competitive alien species) or a lack of co‐evolutionary history with the native community (e.g. Buckley & Catford, 2016; Sheppard & Schurr, 2019). Furthermore, the trajectories of change (cf. Figs 6, 7, 8) in biotic resistance may depend on such different starting conditions. Indeed, shared co‐evolutionary history has been shown to shape competitive dynamics and coexistence (Germain, Weir & Gilbert, 2016). Without explicitly incorporating temporal dynamics (see below), differences between biotic resistance to alien plant invasions and general community assembly processes remain poorly characterised.

Finally, we point out that we only considered the biotic resistance of native species and communities. However, nowadays most communities also include alien plants, herbivores or pathogens. Thus, another open question is to what extent previous alien invasions may modify biotic resistance. Previous invaders may reduce native diversity and with it indirectly biotic resistance or directly compete with or facilitate previously established alien plants, resulting in an ‘invasional meltdown’ (Simberloff & Von Holle, 1999). For instance, being more similar to previous invaders increased the establishment success of alien plants in French grasslands (Sheppard *et al*., 2018), and previous invaders modified the soil microbiome so that the belowground antagonists facilitated aliens over natives (Zhang *et al*., 2020; Table 4).

### (4) Research gaps: temporal dynamics of biotic resistance

Most experimental studies are short‐term and thus do not consider the temporal dynamics of biotic resistance. Temporal dynamics occur at two levels: within the lifetime of individual invaders or competitors/antagonists, and over multiple generations/evolutionary timescales. For example, herbivory may affect early seedling survival (Brian *et al*., 2024) but is less likely to affect the survival of adult plants (Levine *et al*., 2004). Also, effects of high native diversity on biomass of the introduced *Solidago canadensis* increased from the first to the second year of growth, likely due to the accumulation of allelochemicals in the second year (Wang *et al*., 2025). Ecological temporal dynamics can also be driven by antagonists learning throughout their lifetime, or phenotypic plasticity (e.g. Phillips & Leger, 2015). For example, herbivorous Mediterranean fish learn to feed on the invasive alga *Caulerpa cylindracea* (Santamaría *et al*., 2022). However, the commonly used snapshot experiments considering a single life‐history stage of an invader (and community) inadequately capture biotic resistance dynamics (Agrawal & Maron, 2022), also reinforcing the need for multiple performance measures.

Evolutionary dynamics of biotic resistance are particularly important for long‐term invader establishment and spread yet remain poorly understood. From the 240 studies in our meta‐analysis, very few studied such temporal dynamics (e.g. Ender, Christian & Cushman, 2017; Eastburn *et al*., 2018; Brendel *et al*., 2023). However, we note that studies focusing on the evolution of native species (e.g. Germain *et al*., 2020; Table 4), without measuring invader performance, were not included in our meta‐analysis, although they are part of the processes depicted in Fig. 5. Indeed, long‐term changes in biotic resistance may be a result of interspecific or intraspecific selection (microevolution), either in the invader or the native species (Figs 5 and 8). In particular, intra‐ and interspecific variation might be more important than total native density in determining possible biotic resistance in the later stages of invasion (Fig. 8). Despite its importance, interspecific selection is probably less often explicitly considered than intraspecific selection (but see Brendel, Schurr & Sheppard, 2021). Moreover, evolutionary shifts in invader populations over time (Gruntman & Segev, 2024), while frequently studied in relation to abiotic changes (e.g. Colautti & Barrett, 2013; Table 4), are rarely studied in the context of biotic resistance. Such intraspecific changes in the invader can result from an effect of the native community at either an ecological timescale (e.g. increased herbivore pressure) or an evolutionary timescale (e.g. selection for increased allocation to defence at the expense of reproduction). Furthermore, intraspecific self‐limiting processes and evolutionary limitations may play a role (Lankau *et al*., 2009; Table 4). For example, garlic mustard (*Alliaria petiolata*), invasive in North America, increased in cover before declining again (Blossey *et al*., 2021). The authors suggested that the reason for these later declines is the accumulation of intraspecifically‐generated negative plant–soil feedback. These results match trends of invasive species in New Zealand, which experienced negative plant–soil feedback 100–300 years after invasion (Diez *et al*., 2010).

The strength of biotic resistance exerted by native communities can depend on the genetic diversity of the resident native species (Fig. 7; Agneray *et al*., 2023; but see Lyberger *et al*., 2025). The empirical evidence suggests that native species can evolve to become more competitive in the presence of invaders, providing stronger biotic resistance over longer timescales (Oduor, 2013; Germain *et al*., 2020) and potentially limiting further invasive spread. Due to such microevolutionary changes, as well as shifts in the composition of the native community, the variation in the native community can become increasingly important in its contribution to biotic resistance over time, as indicated for antagonists in our mathematical model (Fig. 8). In our mathematical model (Section IV.2) we modelled antagonism rather than competition, as our goal was to provide a simple and analytically tractable example of how biotic resistance could change over time. We anticipate that the predicted dynamics would be qualitatively similar for competition. Moreover, under the joint action of competition and antagonism evolutionary dynamics are likely to be accelerated given the additional selective pressure that alien plant species exert on native competitors (Oduor, 2013). Modifying our model of biotic resistance dynamics to represent competition as well as the joint action of competition and antagonism would thus be a valuable avenue of future work.

In our review, using country‐ or population‐level residence time as a proxy, we found that biotic resistance weakened until approximately 200 years after invasion, then became stronger again (Fig. 4). This parabolic curve, with a reduction in the effectiveness of biotic resistance at intermediate timescales followed by increased effectiveness at later timescales, is also consistent with predictions of our mathematical model (Fig. 8) and supported by previous hypotheses (Brendel *et al*., 2023). However, the overall non‐linear pattern might also arise due to different relationships between residence time and biotic resistance for different habitat types (Fig. S3). Furthermore, other empirical studies using residence time as a proxy for evolutionary experience have shown consistent increases in the strength of biotic resistance (Sheppard & Schurr, 2019) or no relationship between naïve native communities and those with evolutionary experience of the invader (Brendel *et al*., 2023). Besides the paucity of studies, one problem is that residence time as a proxy may be confounded by non‐random invasion patterns caused by, for example, human selection to introduce particular plants with specific traits (Kinlock *et al*., 2022; Bartlett *et al*., 2023). Accordingly, reduced invader performance with increasing residence time may be due to an increase in biotic resistance by native communities or due to a negative correlation between residence time and competitive ability of alien species (Sheppard & Schurr, 2019). With the available evidence to date, it is difficult to project how biotic resistance will change over time in a specific setting, and what shape the temporal trajectory takes under different conditions.

To piece together the complex dynamics of biotic resistance, a better understanding of key ecological and evolutionary processes linking invasiveness and invasibility is needed (Fig. 5). Future experiments can be specifically designed to focus on individual causal links (see examples in Table 4) to elucidate their relative roles in driving biotic resistance over longer timescales. Suitable approaches include the manipulation of biotic resistance mechanisms in studies comparing naïve (allopatric) *versus* experienced (sympatric) native populations (e.g. Germain *et al*., 2020; Table 4), the impact of invasions of populations of an alien species with varying lengths of residence times (e.g. Lankau *et al*., 2009; Table 4), or as a proxy, distance to source (core *versus* edge) populations of invaders (e.g. Huang & Peng, 2016; Table 4). Another approach is to use multi‐species comparisons of invasion success for species differing in residence time (e.g. Diez *et al*., 2010; Brendel *et al*., 2023). Smaller experiments targeting specific mechanisms should then be complemented by larger, ambitious experiments aimed at generalisations. This can involve large‐scale seed addition studies in which propagules of many species are added to many different community types, following establishment success (and ideally, population dynamics) over time (e.g. Freitag *et al*., 2023). Additionally, observational studies that use the increasingly available long‐term ecological data, such as data from sites that have been repeatedly surveyed for vegetation (e.g. Jandt *et al*., 2022) can be used to examine changes in composition and abundance of aliens over time.

## VI. CONCLUSIONS

(1) Biotic resistance, the reduction in invader performance caused by the native community, is a concept of utmost importance for understanding the long‐term dynamics of plant invasions. As close to four decades of empirical research have shown, native plant competitors and antagonists as well as native community diversity have the capacity to reduce various alien plant performance measures in an invaded community.
(2) Yet, much remains unknown about the interplay of multiple mechanisms of biotic resistance on alien plant performance, particularly population dynamics. Because few studies compare biotic resistance to alien and native species in the same experimental setting, we also do not yet understand the extent to which the effects and responses depend on species origin *versus* identity.
(3) Crucially, the role of biotic resistance in limiting long‐term alien establishment and spread requires further theoretical and empirical attention. Biotic resistance may change over ecological and evolutionary timescales due to a complex series of processes, involving both interspecific and intraspecific selection in alien species and native communities, and aspects of species invasiveness and community invasibility.
(4) To better understand these key processes, temporal dynamics of biotic resistance should be empirically studied by manipulating biotic resistance mechanisms in the field, monitoring recipient invaded communities over multiple generations, comparing communities exposed to the same invaders for varying lengths of time or with varying distances from source populations, or comparing different invader species with varying residence times. Thereby, both inter‐ and intraspecific selection in alien and native species must be considered to elucidate their relative roles in driving biotic resistance over long timescales.
(5) Beyond its importance in understanding fundamental ecological and evolutionary processes, biotic resistance presents an essential tool for ecological restoration and conservation of biodiversity. Once a better understanding of its dynamics is achieved, long‐term invasion management may be significantly improved.

## Supporting information


**Appendix S1.** List of studies included in the meta‐analysis.
**Fig. S1.** Distribution of the 1468 observations from 240 studies on alien plant performance according to biotic resistance mechanism, performance measure and various moderators.
**Fig. S2.** Results of a phylogenetic model without an interaction between biotic resistance mechanism and performance measure.
**Fig. S3.** Effect of residence time of alien plant species on the magnitude of biotic resistance (Hedges' *g*) to plant invasions.
**Table S1.** List of most common alien species.
**Table S2.** Meta‐analysis models, shown with their significance tests.
**Table S3.** Marginal means for individual moderators in the meta‐analysis.
**Table S4.** The biotic resistance mechanism combinations with the numbers of effect sizes with additive, synergistic, antagonistic and reversal effects for each.
**Table S5.** Effect of residence time depending on other moderators.
**Appendix S2.** A mathematical model for the temporal dynamics of biotic resistance by native antagonists.
**Table S6.** Model parameters that were kept constant in all scenarios.
**Table S7.** Alternative scenarios for biotic resistance and corresponding parameter settings.

## Data Availability

Data and R code to reproduce the meta‐analysis and model are publicly available at DRYAD: https://doi.org/10.5061/dryad.c2fqz61qr.
