# Supplementary material for: Dynamics of biotic resistance to plant invasions
Source: Biol Rev Camb Philos Soc. 2026 Jun 3;101(6):2554–78. doi: 10.1002/brv.70190 (PMC13736819; doi:10.1002/brv.70190)
Supplement: Supplementary file 1 [file BRV-101-2554-s001.pdf]

Supporting information: dynamics of biotic resistance to plant invasions

Christine S. Sheppard, Joshua I. Brian, Wayne Dawson, Petr Dostál, Franz Essl, Michal Gruntman, Tina Heger, Mark van Kleunen, Mialy Razanajatovo, Lena C. Reimann, Frank M. Schurr, Montserrat Vilà, Florencia A. Yannelli and Marina Golivets

Appendix S1. List of studies included in the meta-analysis

Studies investigating combined effects of two biotic resistance mechanisms are highlighted in bold (but note that studies 86, 111, 144, 145, 155 and 161 could not be included in the analysis on combined effects of multiple biotic resistance mechanisms as they only included binomial data in the performance category "survival").

1. ABELLA, S.R., CRAIG, D.J., SMITH, S.D. & NEWTON, A.C. (2012). Identifying native vegetation for reducing exotic species during the restoration of desert ecosystems. *Restoration Ecology* **20**, 781–787. DOI: 10.1111/j.1526-100X.2011.00848.x
2. ADAIR, E.C., BURKE, I.C. & LAUENROTH, W.K. (2008). Contrasting effects of resource availability and plant mortality on plant community invasion by *Bromus tectorum* L. *Plant and Soil* **304**, 103–115. DOI: 10.1007/s11104-007-9525-9
3. ADOMAKO, M.O., NING, L., TANG, M., DU, D.-L., VAN KLEUNEN, M. & YU, F.-H. (2019). Diversity- and density-mediated allelopathic effects of resident plant communities on invasion by an exotic plant. *Plant and Soil* **440**, 581–592. DOI: 10.1007/s11104-019-04123-9

4. AMSBERRY, L., BAKER, M.A., EWANCHUK, P.J. & BERTNESS, M.D. (2000). Clonal integration and the expansion of *Phragmites australis*. *Ecological Applications* **10**, 1110–1118. DOI: 10.1890/1051-0761(2000)010[1110:CIATEO]2.0.CO;2

5. ANDONIAN, K., HIERRO, J.L., KHETSURIANI, L., BECERRA, P.I., JANOYAN, G., VILLAREAL, D., CAVIERES, L.A., FOX, L.R. & CALLAWAY, R.M. (2012). Geographic mosaics of plant-soil microbe interactions in a global plant invasion. *Journal of Biogeography* **39**, 600–608. DOI: 10.1111/j.1365-2699.2011.02629.x

6. ASHTON, I.W. & LERDAU, M.T. (2008). Tolerance to herbivory, and not resistance, may explain differential success of invasive, naturalized, and native North American temperate vines. *Diversity and Distributions* **14**, 169–178. DOI: 10.1111/j.1472-4642.2007.00425.x

7. AVERILL, K.M., MORTENSEN, D.A., SMITHWICK, E.A.H., KALISZ, S., MCSHEA, W.J., BOURG, N.A., PARKER, J.D., ROYO, A.A., ABRAMS, M.D., APSLEY, D.K., BLOSSEY, B., BOUCHER, D.H., CARAHER, K.L., DITOMMASO, A., JOHNSON, S.E., MASSON, R. & NUZZO, V.A. (2018). A regional assessment of white-tailed deer effects on plant invasion. *AoB PLANTS* **10**, plx047. DOI: 10.1093/aobpla/plx047

8. BAKKER, J. & WILSON, S. (2001). Competitive abilities of introduced and native grasses. *Plant Ecology* **157**, 119–127. DOI: 10.1023/A:1013972403293

9. BALESTRI, E., VALLERINI, F., MENICAGLI, V., BARNABA, S. & LARDICCI, C. (2018). Biotic resistance and vegetative propagule pressure co-regulate the invasion success of a marine clonal macrophyte. *Scientific Reports* **8**, 16621. DOI: 10.1038/s41598-018-35015-0

10. BARGER, N.N., D'ANTONIO, C.M., GHNEIM, T. & CUEVAS, E. (2003). Constraints to colonization and growth of the African grass, *Melinis minutiflora*, in a Venezuelan savanna. *Plant Ecology* **167**, 31–43. DOI: 10.1023/A:1023903901286

11. BATISTELI, A.F., COSTA, R.O. & CHRISTIANINI, A.V. (2020). Seed abundance affects seed removal of an alien and a native tree in the Brazilian savanna: Implications for biotic resistance. *Austral Ecology* **45**, 1007–1015. DOI: 10.1111/aec.12922

12. BAYLISS, S.L.J., TERHORST, C.P. & LAU, J.A. (2017). Testing genotypic variation of an invasive plant species in response to soil disturbance and herbivory. *Oecologia* **183**, 1135–1141. DOI: 10.1007/s00442-017-3820-9

13. BECERRA, P.I. & BUSTAMANTE, R.O. (2011). Effect of a native tree on seedling establishment of two exotic invasive species in a semiarid ecosystem. *Biological Invasions* **13**, 2763–2773. DOI: 10.1007/s10530-011-9961-6

14. BECKSTEAD, J. & PARKER, I.M. (2003). Invasiveness of *Ammophila arenaria*: release from soil-borne pathogens? *Ecology* **84**, 2824–2831. DOI: 10.1890/02-0517

15. BELL, M.D., LULOW, M.E., BALAZS, K.R., HUXMAN, K.A., MCCOLLUM, J.R., HUXMAN, T.E. & KIMBALL, S. (2019). Restoring a Mediterranean-climate shrub community with perennial species reduces future invasion. *Restoration Ecology* **27**, 298–307. DOI: 10.1111/rec.12851

16. BELLINGHAM, P.J. & COOMES, D.A. (2003). Grazing and community structure as determinants of invasion success by Scotch broom in a New Zealand montane shrubland. *Diversity and Distributions* **9**, 19–28. DOI: 10.1046/j.1472-4642.2003.00162.x

17. BENNETT, J.A. & PÄRTEL, M. (2021). Using functional dissimilarity among species pools and communities to predict establishment of native and alien species. *Journal of Vegetation Science* **32**, e13062. DOI: 10.1111/jvs.13062

18. BENNETT, J.A., RIIBAK, K., KOOK, E., REIER, Ü., TAMME, R., GUILLERMO BUENO, C. &, PÄRTEL, M. (2016). Species pools, community completeness and invasion: disentangling diversity effects on the establishment of native and alien species. *Ecology Letters* **19**, 1496–1505. DOI: 10.1111/ele.12702

19. Bin, F., Wei, W., Zhu, P., Zhang, J.L., Zhang, P. & Hou, Y.P. (2019). Allelopathic mechanisms in community resistance to exotic plants invasions: allelopathic effects of *Pinus densiflora* Siebold and Zucc. and *P. thunbergia* Parlat. on the exotic invasive plant *Rhus typhina* L. *Allelopathy Journal* **46**, 229–240. DOI: 10.26651/allelo.j/2019-46-2-1211

20. Bishop, T.B.B., Gill, R.A., McMillan, B.R. & St. Clair, S.B. (2020). Fire, rodent herbivory, and plant competition: implications for invasion and altered fire regimes in the Mojave Desert. *Oecologia* 192, 155–167. DOI: 10.1007/s00442-019-04562-2

21. Biswas, S.R., Kotanen, P.M., Kambo, D. & Wagner, H.H. (2015). Context-dependent patterns, determinants and demographic consequences of herbivory in an invasive species. *Biological Invasions* **17**, 165–178. DOI: 10.1007/s10530-014-0715-0

22. Bohl Stricker, K. & Stiling, P. (2014). Release from herbivory does not confer invasion success for *Eugenia uniflora* in Florida. *Oecologia* **174**, 817–826. DOI: 10.1007/s00442-013-2798-1

23. Bossard, C.C. (1991). The role of habitat disturbance, seed predation and ant dispersal on establishment of the exotic shrub *Cytisus scoparius* in California. *American Midland Naturalist* **126**, 1–13. DOI: 10.2307/2426145

24. Brendel, M.R., Schurr, F.M. & Sheppard, C.S. (2023). Alien plant fitness is limited by functional trade-offs rather than a long-term increase in competitive effects of native communities. *Ecology and Evolution* **13**, e10468. DOI: 10.1002/ece3.10468

25. Britton-Simmons, K.H. (2006). Functional group diversity, resource preemption and the genesis of invasion resistance in a community of marine algae. *Oikos* **113**, 395–401. DOI: 10.1111/j.2006.0030-1299.14203.x

26. Britton-Simmons, K.H. & Abbott, K.C. (2008). Short- and long-term effects of disturbance and propagule pressure on a biological invasion. *Journal of Ecology* **96**, 68–77. DOI: 10.1111/j.1365-2745.2007.01319.x

27. BROOKS, W.R. & JORDAN, R.C. (2013). Propagule pressure and native species richness effects drive invasibility in tropical dry forest seedling layers. *Perspectives in Plant Ecology, Evolution and Systematics* **15**, 162–170. DOI: 10.1016/j.ppees.2013.03.001

28. BROWN, C.S. & RICE, K.J. (2010). Effects of belowground resource use complementarity on invasion of constructed grassland plant communities. *Biological Invasions* **12**, 1319–1334. DOI: 10.1007/s10530-009-9549-6

29. BULLERI, F., TAMBURELLO, L. & BENEDETTI-CECCHI, L. (2009). Loss of consumers alters the effects of resident assemblages on the local spread of an introduced macroalga. *Oikos* 118, 269–279. DOI: 10.1111/j.1600-0706.2008.16955.x

30. BUNN, R.A., ANTUNES, P.M., BULLINGTON, L.S., FAHEY, C. & LEKBERG, Y. (2023). Soil moisture and competition determine soil biota effects on invasive *Centaurea stoebe*. *Functional Ecology* **37**, 1181–1196. DOI: 10.1111/1365-2435.14295

31. BYUN, C., CHOI, H. & KANG, H. (2020). Effects of cutting and sowing seeds of native species on giant ragweed invasion and plant diversity in a field experiment. *Journal of Ecology and Environment* **44**, 28. DOI: 10.1186/s41610-020-00173-8

32. BYUN, C., DE BLOIS, S. & BRISSON, J. (2020). Restoring functionally diverse communities enhances invasion resistance in a freshwater wetland. *Journal of Ecology* **108**, 2485–2498. DOI: 10.1111/1365-2745.13419

33. BYUN, C., JEONG, Y. & HONG, S.H. (2022). Synergistic effects of soil nutrient level and native species identity and diversity on biotic resistance to *Sicyos angulatus*, an invasive species. *Oecologia* **200**, 221–230. DOI: 10.1007/s00442-022-05265-x

34. CALLAWAY, R., NEWINGHAM, B., ZABINSKI, C.A. & MAHALL, B.E. (2001). Compensatory growth and competitive ability of an invasive weed are enhanced by soil fungi and native neighbours. *Ecology Letters* 4, 429–433. DOI: 10.1046/j.1461-0248.2001.00251.x

35. Callaway, R.M., Mahall, B.E., Wicks, C., Pankey, J. & Zabinski, C. (2003). Soil fungi and the effects of an invasive forb on grasses: neighbor identity matters. *Ecology* **84**, 129–135. DOI: 10.1890/0012-9658(2003)084[0129:SFATEO]2.0.CO,2

36. Callaway, R.M., Montesinos, D., Williams, K. & Maron, J.L. (2013). Native congeners provide biotic resistance to invasive *Potentilla through* soil biota. *Ecology* **94**, 1223–1229. DOI: 10.1890/12-1875.1

37. Callaway, R.M., Thelen, G.C., Barth, S., Ramsey, P.W. & Gannon, J.E. (2004). Soil fungi alter interactions between the invader *Centaurea maculosa* and North American natives. *Ecology* **85**, 1062–1071. DOI: 10.1890/02-0775

38. Callaway, R.M., Thelen, G.C., Rodriguez, A. & Holben, W.E. (2004). Soil biota and exotic plant invasion. *Nature* **427**, 731–733. DOI: 10.1038/nature02322

39. Calvo, C., Mormul, R.P., Figueiredo, B.R.S., Cunha, E.R., Thomaz, S.M. & Meerhoff, M. (2019). Herbivory can mitigate, but not counteract, the positive effects of warming on the establishment of the invasive macrophyte *Hydrilla verticillata*. *Biological Invasions* **21**, 59–66. DOI: 10.1007/s10530-018-1803-3

40. Caño, L., Escarré, J. & Sans, F.X. (2007). Factors affecting the invasion success of *Senecio inaequidens* and *S. pterophorus* in Mediterranean plant communities. *Journal of Vegetation Science* **18**, 281–288. DOI: 10.1658/1100-9233(2007)18[281:FATISO]2.0.CO,2

41. Caronni, S., Calabretti, C., Delaria, M.A., Bernardi, G., Navone, A., Occhipinti-Ambrogi, A., Panzalis, P. & Ceccherelli, G. (2015). Consumer depletion alters seagrass resistance to an invasive macroalga. *PLOS ONE* **10**, e0115858. DOI: 10.1371/journal.pone.0115858

42. Carrillo, J. & Siemann, E. (2016). A native plant competitor mediates the impact of above-and belowground damage on an invasive tree. *Ecological Applications* **26**, 2060–2071. DOI: 10.1002/eap.1359

43. Carrillo-Gavilán, M.A., Lalaguee, H. & Vila, M. (2010). Comparing seed removal of 16 pine species differing in invasiveness. *Biological Invasions* **12**, 2233–2242. DOI: 10.1007/s10530-009-9633-y

44. Case, C.M. & Crawley, M.J. (2000). Effect of interspecific competition and herbivory on the recruitment of an invasive alien plant: *Conyza sumatrensis*. *Biological Invasions* **2**, 103–110. DOI: 10.1023/A:1010084305933

45. Cebrian, E., Ballesteros, E., Linares, C. & Tomas, F. (2011). Do native herbivores provide resistance to Mediterranean marine bioinvasions? A seaweed example. *Biological Invasions* **13**, 1397–1408. DOI: 10.1007/s10530-010-9898-1

46. Chambers, J.C., Roundy, B.A., Blank, R.R., Meyer, S.E. & Whittaker, A. (2007). What makes Great Basin sagebrush ecosystems invasible by *Bromus tectorum*? *Ecological Monographs* **77**, 117–145. DOI: 10.1890/05-1991

47. Charles, L.S., Maron, J.L. & Larios, L. (2022). Species provenance and traits mediate establishment and performance in an invaded grassland. *Functional Ecology* **36**, 1528–1541. DOI: 10.1111/1365-2435.14044

48. Chen, B.-M., Li, S., Liao, H.-X. & Peng, S.-L. (2017). Do forest soil microbes have the potential to resist plant invasion? A case study in Dinghushan Biosphere Reserve (South China). *Acta Oecologica* **81**, 1–9. DOI: 10.1016/j.actao.2017.04.003

49. Chen, D. & van Kleunen, M. (2022). Competitive effects of plant invaders on and their responses to native species assemblages change over time. *NeoBiota* **73**, 19–37. DOI: 10.3897/neobiota.73.80410

50. Chen, H., Chang, R., Wang, R. & Liu, J. (2012). The biotic resistance of soil biota on the invasive weed coreopsis grandiflora varies with irradiance levels. *Advanced Materials Research* **573–574**, 1044–1051. DOI: 10.4028/www.scientific.net/AMR.573-574.1044

51. CHEN, H., WANG, R.Q., GE, X.L., ZHANG, J., DU, N., WANG, W. & LIU, J. (2012). Competition and soil fungi affect the physiological and growth traits of an alien and a native tree species. *Photosynthetica* **50**, 77–85. DOI: 10.1007/s11099-012-0013-y

52. CHEN, P., HUANG, Q., ZHUGE, Y., LI, C., ZHU, P. & HOU, Y. (2021). The effects of plant–soil feedback on invasion resistance are soil context dependent. *Oecologia* **197**: 213–222. DOI: 10.1007/s00442-021-05004-8

53. CHEN, P.-D., HOU, Y.-P., ZHUGE, Y.-H., WEI, W. & HUANG, Q.-Q. (2019). The effects of soils from different forest types on the growth of the invasive plant *Phytolacca americana*. *Forests* **10**, 492. DOI: 10.3390/f10060492

54. CHRISTINA, M., ROUIFED, S., PUIJALON, S., VALLIER, F., MEIFFREN, G., BELLVERT, F. & PIOLA, F. (2015). Allelopathic effect of a native species on a major plant invader in Europe. *Science of Nature* **102**, 12. DOI: 10.1007/s00114-015-1263-x

55. CLAIR, S.B.S., O'CONNOR, R., GILL, R. & MCMILLAN, B. (2016). Biotic resistance and disturbance: Rodent consumers regulate post-fire plant invasions and increase plant community diversity. *Ecology* **97**, 1700–1711. DOI: 10.1111/ecy.2016.97.issue-7

56. CONNOLLY, B.M., CARRIS, L.M. & MACK, R.N. (2018). Soil-borne seed pathogens: contributors to the naturalization gauntlet in Pacific Northwest (USA) forest and steppe communities? *Plant Ecology* **219**, 359–368. DOI: 10.1007/s11258-018-0800-z

57. CONNOLLY, B.M., PEARSON, D.E. & MACK, R.N. (2014). Granivory of invasive, naturalized, and native plants in communities differentially susceptible to invasion. *Ecology* **95**, 1759–1769. DOI: 10.1890/13-1774.1

58. CONNOLLY, B.M., POWERS, J. & MACK, R.N. (2017). Biotic constraints on the establishment and performance of native, naturalized, and invasive plants in Pacific Northwest (USA) steppe and forest. *NeoBiota* **34**, 21–40. DOI: 10.3897/neobiota.34.10820

59. COOKE, M.M., MARTELLI, A., SLEIMAN, M. & CIPRIOTTI, P.A. (2021). The role of competition on invader colonization along stress gradients in the Fuegian steppe. *Oecologia* **195**, 1031–1040. DOI: 10.1007/s00442-021-04894-y

60. CRANDALL, R.M. & KNIGHT, T.M. (2018). Role of multiple invasion mechanisms and their interaction in regulating the population dynamics of an exotic tree. *Journal of Applied Ecology* **55**, 885–894. DOI: 10.1111/1365-2664.13020

61. CUSHMAN, J.H., LORTIE, C.J. & CHRISTIAN, C.E. (2011). Native herbivores and plant facilitation mediate the performance and distribution of an invasive exotic grass. *Journal of Ecology* **99**, 524–531. DOI: 10.1111/j.1365-2745.2010.01776.x

62. DE LA PEÑA, E., DE CLERCQ, N., BONTE, D., ROILOA, S., RODRÍGUEZ-ECHEVERRÍA, S. & FREITAS, H. (2010). Plant-soil feedback as a mechanism of invasion by *Carpobrotus edulis*. *Biological Invasions* **12**, 3637–3648. DOI: 10.1007/s10530-010-9756-1

63. DE VILLALOBOS, A.E., ZALBA, S.M. & PELÁEZ, D.V. (2011). *Pinus halepensis* invasion in mountain pampean grassland: effects of feral horses grazing on seedling establishment. *Environmental Research* **111**, 953–959. DOI: 10.1016/j.envres.2011.03.011

64. DING, J. &, BLOSSEY, B. (2005). Impact of the native water lily leaf beetle, *Galerucella nymphaeae* (Coleoptera: Chrysomelidae), attacking introduced water chestnut, *Trapa natans*, in the northeastern United States. *Environmental Entomology* **34**, 683–689. DOI: 10.1603/0046-225X-34.3.683

65. DOSTÁL, P. & PALEČKOVÁ, M. (2011). Does relatedness of natives used for soil conditioning influence plant-soil feedback of exotics? *Biological Invasions* **13**, 331–340. DOI: 10.1007/s10530-010-9824-6

66. DU, X.-D., WANG, J., SHEN, C., WANG, J., JING, Z., HUANG, L.-N., LUO, Z.-H. & GE, Y. (2023). Increased leaf bacterial network complexity along the native plant diversity gradient facilitates plant Invasion? *Plants* **12**, 1406. DOI: 10.3390/plants12061406

67. DUKES, J.S. (2002). Species composition and diversity affect grassland susceptibility and response to invasion. *Ecological Applications* **12**: 602–617. DOI: 10.1890/1051-0761(2002)012[0602:SCADAG]2.0.CO,2

68. EASTBURN, D.J., ROCHE, L.M., DORAN, M.P., BLAKE, P.R., BOURIL, C.S., GAMBLE, G. & GORNISH, E.S. (2018). Seeding plants for long-term multiple ecosystem service goals. *Journal of Environmental Management* **211**, 191–197. DOI: 10.1016/j.jenvman.2018.01.061

69. ECKBERG, J.O., TENHUMBERG, B. & LOUDA, S.M. (2012). Insect herbivory and propagule pressure influence *Cirsium vulgare* invasiveness across the landscape. *Ecology* **93**, 1787–1794. DOI: 10.1890/11-1583.1

70. ECKBERG, J.O., TENHUMBERG, B. & LOUDA, S.M. (2014). Native insect herbivory limits population growth rate of a non-native thistle. *Oecologia* **175**, 129–138. DOI: 10.1007/s00442-013-2876-4

71. EL-BAROUGY, R., MACIVOR, J.S., ARNILLAS, C.A., NADA, R.M., KHEDR, A.-H.A. & CADOTTE, M.W. (2020). Richness, phylogenetic diversity, and abundance all have positive effects on invader performance in an arid ecosystem. *Ecosphere* **11**, e03045. DOI: 10.1002/ecs2.3045

72. ENDER, C.L., CHRISTIAN, C.E. & CUSHMAN, J.H. (2017). Native herbivores and environmental heterogeneity as mediators of an exotic grass invasion. *Ecology and Evolution* **7**, 1561–1571. DOI: 10.1002/ece3.2727

73. ERNEBERG, M. (1999). Effects of herbivory and competition on an introduced plant in decline. *Oecologia* 118, 203–209. DOI: 10.1007/s004420050719

74. ESKELINEN, A. & HARRISON, S. (2014). Exotic plant invasions under enhanced rainfall are constrained by soil nutrients and competition. *Ecology* **95**, 682–692. DOI: 10.1890/13-0288.1

75. EVANGELISTA, H.B., MICHELAN, T.S., GOMES, L.C. & THOMAZ, S.M. (2017). Shade provided by riparian plants and biotic resistance by macrophytes reduce the establishment of an invasive Poaceae. *Journal of Applied Ecology* **54**, 648–656. DOI: 10.1111/1365-2664.12791

76. FAN, S., YU, D. & LIU, C. (2013). The invasive plant *Alternanthera philoxeroides* was suppressed more intensively than its native congener by a native generalist: implications for the biotic resistance hypothesis. *PLOS ONE* **8**, e83619. DOI: 10.1371/journal.pone.0083619

77. FAN, S., YU, H., DONG, X., WANG, L., CHEN, X., YU, D. & LIU, C. (2016). Invasive plant *Alternanthera philoxeroides* suffers more severe herbivory pressure than native competitors in recipient communities. *Scientific Reports* **6**, 36542. DOI: 10.1038/srep36542

78. FENG, Y., FOUQUERAY, T.D. &, VAN KLEUNEN, M. (2019). Linking Darwin's naturalisation hypothesis and Elton's diversity–invasibility hypothesis in experimental grassland communities. *Journal of Ecology* **107**, 794–805. DOI: 10.1111/1365-2745.13061

79. GAO, F.-L., HE, Q.-S., XIE, R.-Q., HOU, J.-H., SHI, C.-L., LI, J.-M. & YU, F.-H. (2021). Interactive effects of nutrient availability, fluctuating supply, and plant parasitism on the post-invasion success of Bidens pilosa. *Biological Invasions* 23, 3035–3046. DOI: 10.1007/s10530-021-02555-y

80. GAO, L., ZAN, Q.J., LI, M.G., GUO, Q., HU, L., JIANG, L., ZHOU, S. & LIU, H.J. (2013). Soil fungi of three native tree species inhibit biomass production and shift biomass allocation of invasive *Mikania micrantha* Kunth. *Chinese Science Bulletin* **58**, 758–765. DOI: 10.1007/s11434-012-5394-6

81. GELBARD, J.L. &, HARRISON, S. (2005). Invasibility of roadless grasslands: an experimental study of yellow starthistle. *Ecological Applications* **15**, 1570–1580. DOI: 10.1890/03-5362

82. GOING, B.M., HILLERISLAMBERS, J. & LEVINE, J.M. (2009). Abiotic and biotic resistance to grass invasion in serpentine annual plant communities. *Oecologia* **159**, 839–847. DOI: 10.1007/s00442-008-1264-y

83. GREEN, P.T., LAKE, P.S. & O'DOWD, D.J. (2004). Resistance of island rainforest to invasion by alien plants: influence of microhabitat and herbivory on seedling performance. *Biological Invasions* 6, 1–9. DOI: 10.1023/B:BINV.0000010144.12808.cb

84. GRIBBEN, P.E., NIELSEN, S., SEYMOUR, J.R., BRADLEY, D.J., WEST, M.N. & THOMAS, T. (2017). Microbial communities in marine sediments modify success of an invasive macrophyte. *Scientific Reports* **7**, 9845. DOI: 10.1038/s41598-017-10231-2

85. GRIBBEN, P.E., THOMAS, T., PUSCEDDU, A., BONECHI, L., BIANCHELLI, S., BUSCHI, E., NIELSEN, S., RAVAGLIOLI, C. & BULLERI, F. (2018). Below-ground processes control the success of an invasive seaweed. *Journal of Ecology* **106**, 2082–2095. DOI: 10.1111/1365-2745.12966

86. GRIESHOP, M.J. & NOWIERSKI, R.M. (2002). Selected factors affecting seedling recruitment of dalmatian toadflax. *Journal of Range Management* 55, 612–619. DOI: 10.2307/4004005

87. GUYTON, J.A., PANSU, J., HUTCHINSON, M.C., KARTZINEL, T.R., POTTER, A.B., COVERDALE, T.C., DASKIN, J.H., DA CONCEIÇÃO, A.G., PEEL, M.J.S., STALMANS, M.E. &, PRINGLE, R.M. (2020). Trophic rewilding revives biotic resistance to shrub invasion. *Nature Ecology and Evolution* **4**, 712–724. DOI: 10.1038/s41559-019-1068-y

88. HAMMER, C.F. & GUNN, J.S. (2021). Planting native trees to restore riparian forests increases biotic resistance to nonnative plant invasions. *Invasive Plant Science and Management* **14**, 126–133. DOI: 10.1017/inp.2021.11

89. HANNAM, M.P. & WYLLIE-ECHEVERRIA, S. (2015). Microtopography promotes coexistence of an invasive seagrass and its native congener. *Biological Invasions* **17**, 381–395. DOI: 10.1007/s10530-014-0736-8

90. HARMS, N.E. (2020). Competitive interactions of flowering rush (*Butomus umbellatus* L.) cytotypes in submersed and emergent experimental aquatic plant communities. *Diversity* **12**, 40. DOI: 10.3390/d12010040

91. HAVRILLA, C.A. & BARGER, N.N. (2018). Biocrusts and their disturbance mediate the recruitment of native and exotic grasses from a hot desert ecosystem. *Ecosphere* **9**, e02361. DOI: 10.1002/ecs2.2361

92. HECKMAN, R.W. & CARR, D.E. (2016). Effects of soil nitrogen availability and native grass diversity on exotic forb dominance. *Oecologia* **182**, 803–813. DOI: 10.1007/s00442-016-3692-4

93. HECKMAN, R.W., HALLIDAY, F.W., WILFAHRT, P.A. & MITCHELL, C.E. (2017). Effects of native diversity, soil nutrients, and natural enemies on exotic invasion in experimental plant communities. *Ecology* 98, 1409–1418. DOI: 10.1002/ecy.1796

94. HERRERA, L.P. & LATERRA, P. (2009). Do seed and microsite limitation interact with seed size in determining invasion patterns in flooding Pampa grasslands? *Plant Ecology* **201**, 457–469. DOI: 10.1007/s11258-008-9542-7

95. HIERRO, J.L., LORTIE, C.J., VILLARREAL, D., ESTANGA-MOLLICA, M.E. & CALLAWAY, R.M. (2011). Resistance to *Centaurea solstitialis* invasion from annual and perennial grasses in California and Argentina. *Biological Invasions* **13**, 2249–2259. DOI: 10.1007/s10530-011-0037-4

96. HIERRO, J.L., VILLARREAL, D., EREN, Ö., GRAHAM, J.M. & CALLAWAY, R.M. (2006). Disturbance facilitates invasion: the effects are stronger abroad than at home. *American Naturalist* **168**, 144–156. DOI: 10.1086/505767

97. HOOPER, D.U. & DUKES, J.S. (2010). Functional composition controls invasion success in a California serpentine grassland. *Journal of Ecology* **98**, 764–777. DOI: 10.1111/j.1365-2745.2010.01673.x

98. HOU, M. & WANG, J. (2023). Functional traits of both specific alien species and receptive community but not community diversity determined the invasion success under biotic and abiotic conditions. *Functional Ecology* **37**, 2598–2610. DOI: 10.1111/1365-2435.14411

99. HUANG, W., CARRILLO, J., DING, J. & SIEMANN, E. (2012). Interactive effects of herbivory and competition intensity determine invasive plant performance. *Oecologia* **170**, 373–382. DOI: 10.1007/s00442-012-2328-6

100. JAUNI, M. & RAMULA, S. (2017). Demographic mechanisms of disturbance and plant diversity promoting the establishment of invasive *Lupinus polyphyllus*. *Journal of Plant Ecology* **10**, 510–517. DOI: 10.1093/jpe/rtw049

101. JESSON, L., KELLY, D. & SPARROW, A. (2000). The importance of dispersal, disturbance, and competition for exotic plant invasions in Arthur's Pass National Park, New Zealand. *New Zealand Journal of Botany* **38**, 451–468. DOI: 10.1080/0028825X.2000.9512696

102. JIN, H., CHANG, L., VAN KLEUNEN, M. & LIU, Y. (2022). Soil mesofauna may buffer the negative effects of drought on alien plant invasion. *Journal of Ecology* **110**, 2332-2342. DOI: 10.1111/1365-2745.13950

103. KALISZ, S., SPIGLER, R.B. & HORVITZ, C.C. (2014). In a long-term experimental demography study, excluding ungulates reversed invader's explosive population growth rate and restored natives. *Proceedings of the National Academy of Sciences of the United States of America* **111**, 4501–4506. DOI: 10.1073/pnas.1310121111

104. KATZ, D.S.W. & IBÁÑEZ, I. (2017). Differences in biotic interactions across range edges have only minor effects on plant performance. *Journal of Ecology* **105**, 321–331. DOI: 10.1111/1365-2745.12675

105. KERNS, B.K. & DAY, M.A. (2014). Fuel reduction, seeding, and vegetation in a juniper woodland. *Rangeland Ecology and Management* **67**, 667–679. DOI: 10.2111/REM-D-13-00149.1

106. KETTENRING, K.M., WHIGHAM, D.F., HAZELTON, E.L.G., GALLAGHER, S.K. & WEINER, H.M. (2015). Biotic resistance, disturbance, and mode of colonization impact the invasion of a widespread, introduced wetland grass. *Ecological Applications* **25**, 466–480. DOI: 10.1890/14-0434.1

107. KNAPP, L.B., FOWNES, J.H. & HARRINGTON, R.A. (2008). Variable effects of large mammal herbivory on three non-native versus three native woody plants. *Forest Ecology and Management* 255, 92–98. DOI: 10.1016/j.foreco.2007.08.023

108. KORELL, L., LANG, B.R., HENSEN, I., AUGE, H. & BRUELHEIDE, H. (2017). Interactions count: plant origin, herbivory and disturbance jointly explain seedling recruitment and community structure. *Scientific Reports* **7**, 8288. DOI: 10.1038/s41598-017-08401-3

109. KORELL, L., STEIN, C., HENSEN, I., BRUELHEIDE, H., SUDING, K.N. & AUGE, H. (2016). Stronger effect of gastropods than rodents on seedling establishment, irrespective of exotic or native plant species origin. *Oikos* **125**, 1467–1477. DOI: 10.1111/oik.02696

110. LAMBRINOS, J.G. (2002). The variable invasive success of *Cortaderia* species in a complex landscape. *Ecology* **83**, 518–529. DOI: 10.1890/0012-9658(2002)083[0518:TVISOC]2.0.CO,2

111. LAMBRINOS, J.G. (2006). Spatially variable propagule pressure and herbivory influence invasion of chaparral shrubland by an exotic grass. *Oecologia* 147, 327–334. DOI: 10.1007/s00442-005-0259-1

112. LAVOREL, S., PRIEUR-RICHARD, A.-H. & GRIGULIS, K. (1999). Invasibility and diversity of plant communities: from patterns to processes. *Diversity and Distributions* **5**, 41–49. DOI: 10.1046/j.1472-4642.1999.00034.x

113. LEFFLER, A.J., LEONARD, E.D., JAMES, J.J. & MONACO, T.A. (2014). Invasion is contingent on species assemblage and invasive species identity in experimental rehabilitation plots. *Rangeland Ecology and Management* **67**, 657–666. DOI: 10.2111/REM-D-13-00140.1

114. LEVINE, J.M. (2000). Species diversity and biological invasions: relating local process to community pattern. *Science* **288**: 852–854. DOI: 10.1126/science.288.5467.852

115. LEVINE, J.M. (2001). Local interactions, dispersal, and native and exotic plant diversity along a California stream. *Oikos* **95**, 397–408. DOI: 10.1034/j.1600-0706.2001.950304.x

116. LI, J., ODUOR, A.M.O., YU, F. & DONG, M. (2019). A native parasitic plant and soil microorganisms facilitate a native plant co-occurrence with an invasive plant. *Ecology and Evolution* 9, 8652–8663. DOI: 10.1002/ece3.5407

117. LI, S.-P., JIA, P., FAN, S.-Y., WU, Y., LIU, X., MENG, Y., LI, Y., SHU, W.-S., LI, J.-T. & JIANG, L. (2022). Functional traits explain the consistent resistance of biodiversity to plant invasion under nitrogen enrichment. *Ecology Letters* **25**, 778–789. DOI: 10.1111/ele.13951

118. LI, Z., WANG, W. & ZHANG, Y. (2014). Recruitment and herbivory affect spread of invasive *Spartina alterniflora* in China. *Ecology* **95**, 1972–1980. DOI: 10.1890/13-2283.1

119. LIANG, A.J., STEIN, C., PEARSON, E., MYERS, J.A., CRANDALL, R.M. & MANGAN, S.A. (2019). Snail herbivory affects seedling establishment in a temperate forest in the Ozarks. *Journal of Ecology* **107**, 1828–1838. DOI: 10.1111/1365-2745.13150

120. LINDIG-CISNEROS, R. & ZEDLER, J.B. (2002). *Phalaris arundinacea* seedling establishment: effects of canopy complexity in fen, mesocosm, and restoration experiments. *Canadian Journal of Botany* **80**, 617–624. DOI: 10.1139/b02-042

121. LIU, L., QUAN, H., DONG, B.C., BU, X.Q., LI, L., LIU, F.D., LEI, G.C. & LI, H.L. (2016). Nutrient enrichment alters impacts of *Hydrocotyle vulgaris* invasion on native plant communities. *Scientific Reports* **6**, 39468. DOI: 10.1038/srep39468

122. LU, X., HE, M., TANG, S., WU, Y., SHAO, X., WEI, H., SIEMANN, E. & DING, J. (2019). Herbivory may promote a non-native plant invasion at low but not high latitudes. *Annals of Botany* **124**, 819–827. DOI: 10.1093/aob/mcz121

123. LU, X.M., SHAO, X. & DING, J.Q. (2014). No impact of a native beetle on exotic plant performance and competitive ability due to plant compensation. *Plant Ecology* 215, 275–284. DOI: 10.1007/s11258-014-0296-0

124. LUCERO, J.E. & CALLAWAY, R.M. (2018). Native granivores reduce the establishment of native grasses but not invasive *Bromus tectorum*. *Biological Invasions* **20**, 3491–3497. DOI: 10.1007/s10530-018-1789-x

125. LUCERO, J.E., SCHAFFNER, U., ASADI, G., BAGHERI, A., RAJABOV, T. & CALLAWAY, R.M. (2019). Enemy release from the effects of generalist granivores can facilitate *Bromus tectorum* invasion in the Great Basin Desert. *Ecology and Evolution* **9**, 8490–8499. DOI: 10.1002/ece3.5314

126. LYONS, K.G. & SCHWARTZ, M.W. (2001). Rare species loss alters ecosystem function - Invasion resistance. *Ecology Letters* **4**, 358–365. DOI: 10.1046/j.1461-0248.2001.00235.x

127. MALECORE, E.M., BERTHELOT, S., VAN KLEUNEN, M. & RAZANAJATOVO, M. (2021). Reciprocal heterospecific pollen interference among alien and native species. *Perspectives in Plant Ecology, Evolution and Systematics* **50**, 125610. DOI: 10.1016/j.ppees.2021.125610

128. MAO, R., LU, X. & DING, J. (2011). Effects of a nematode *Meloidogyne incognita* and its interaction with above-ground herbivory on an invasive wetland plant, alligator weed (*Alternanthera philoxeroides*). *Plant Species Biology* 26, 73–83. DOI: 10.1111/j.1442-1984.2010.00309.x

129. MARON, J. & MARLER, M. (2007). Native plant diversity resists invasion at both low and high resource levels. *Ecology* **88**, 2651–2661. DOI: 10.1890/06-1993.1

130. MARON, J.L., AUGE, H., PEARSON, D.E., KORELL, L., HENSEN, I., SUDING, K.N. & STEIN, C. (2014). Staged invasions across disparate grasslands: effects of seed provenance, consumers and disturbance on productivity and species richness. *Ecology Letters* 17, 499–507. DOI: 10.1111/ele.12250

131. MASON, T.J., FRENCH, K. & JOLLEY, D. (2013). Arrival order among native plant functional groups does not affect invasibility of constructed dune communities. *Oecologia* **173**, 557–568. DOI: 10.1007/s00442-013-2628-5

132. MASON, T.J., FRENCH, K. & JOLLEY, D.F. (2017). Functional richness and identity do not strongly affect invasibility of constructed dune communities. *PLOS ONE* **12**, e0169243. DOI: 10.1371/journal.pone.0169243

133. MATTINGLY, W.B. & REYNOLDS, H.L. (2014). Soil fertility alters the nature of plant–resource interactions in invaded grassland communities. *Biological Invasions* **16**, 2465–2478. DOI: 10.1007/s10530-014-0678-1

134. MATTINGLY, W.B., SWEDO, B.L. & REYNOLDS, H.L. (2010). Interactive effects of resource enrichment and resident diversity on invasion of native grassland by *Lolium arundinaceum*. *Plant Ecology* **207**, 203–212. DOI: 10.1007/s11258-009-9665-5

135. MAZIA, N.C., CHANETON, E.J., GHERSA, C.M. & LEÓN, R.J. (2001). Limits to tree species invasion in pampean grassland and forest plant communities. *Oecologia* **128**, 594–602. DOI: 10.1007/s004420100709

136. MAZZOCHINI, G.G., LIRA-MARTINS, D., DE BARROS, F.V., OLIVEIRA, A.C.C., XAVIER, R.O., FURTADO, M.N., VERONA, L.S., VIANI, R.A.G., ROWLAND, L. & OLIVEIRA, R.S. (2024). Effects of grass functional diversity on invasion success by exotic grasses in Cerrado grasslands. *Journal of Applied Ecology* **61**, 271–280. DOI: 10.1111/1365-2664.14561

137. MCCAY, T.S. & MCCAY, D.H. (2009). Processes regulating the invasion of European buckthorn (*Rhamnus cathartica*) in three habitats of the northeastern United States. *Biological Invasions* **11**, 1835–1844. DOI: 10.1007/s10530-008-9362-7

138. MENICAGLI, V., BALESTRI, E., VALLERINI, F., DE BATTISTI, D. & LARDICCI, C. (2021). Plastics and sedimentation foster the spread of a non-native macroalga in seagrass meadows. *Science of the Total Environment* **757**, 143812. DOI: 10.1016/j.scitotenv.2020.143812

139. METLEN, K.L. & CALLAWAY, R.M. (2015). Native North American pine attenuates the competitive effects of a European invader on native grasses. *Biological Invasions* **17**, 1227–1237. DOI: 10.1007/s10530-014-0790-2

140. MÖHRLE, K., REYES-ALDANA, H.E., KOLLMANN, J. & TEIXEIRA, L.H. (2021). Suppression of an invasive native plant species by designed grassland communities. *Plants* **10**, 775. DOI: 10.3390/plants10040775

141. MORRIS, T.L., BARGER, N.N. & CRAMER, M.D. (2015). Competitive resistance of a native shrubland to invasion by the alien invasive tree species, *Acacia cyclops*. *Biological Invasions* **17**, 3563–3577. DOI: 10.1007/s10530-015-0978-0

142. MORRIS, T.L., BARGER, N.N. & CRAMER, M.D. (2022). Generalist indigenous herbivores resist alien tree invasion: *Rhabdomys pumilio* limits establishment of *Acacia cyclops*. *Biological Invasions* **24**, 1427–1437. DOI: 10.1007/s10530-022-02728-3

143. MULAS, M. & BERTOCCI, I. (2016). Devil's tongue weed (*Grateloupia turuturu* Yamada) in northern Portugal: passenger or driver of change in native biodiversity? *Marine Environmental Research* **118**, 1–9. DOI: 10.1016/j.marenvres.2016.04.007

144. MÜLLER, G., HORSTMEYER, L., RÖNNEBURG, T., VAN KLEUNEN, M. & DAWSON, W. (2016). Alien and native plant establishment in grassland communities is more strongly affected by disturbance than above- and below-ground enemies. *Journal of Ecology* 104, 1233–1242. DOI: 10.1111/1365-2745.12601

145. MUSCHETTO, E., CHANETON, E.J., MAZÍA, N., TRIPODI, M.A. & BUSCH, M. (2022). Biotic resistance in a stochastic world: do rodents act as a filter to alien tree invasion in pampean old fields? *Ecological Research* 37, 568–581. DOI: 10.1111/1440-1703.12318

146. NING, Z., XIE, T., LIU, Z., BAI, J. & CUI, B. (2019). Native herbivores enhance the resistance of an anthropogenically disturbed salt marsh to *Spartina alterniflora* invasion. *Ecosphere* **10**, e02565. DOI: 10.1002/ecs2.2565

147. NITSCHKE, N., EBELING, A., ROTTSTOCK, T., SCHERBER, C., MIDDELHOFF, C., CREUTZBURG, S., WEIGELT, A., TSCHARNTKE, T., FISCHER, M. & WEISSER, W.W. (2010). Time course of plant diversity effects on *Centaurea jacea* establishment and the role of competition and herbivory. *Journal of Plant Ecology* 3, 109–121. DOI: 10.1093/jpe/rtp036

148. OAKLEY, C.A. & KNOX, J.S. (2013). Plant species richness increases resistance to invasion by non-resident plant species during grassland restoration. *Applied Vegetation Science* **16**, 21–28. DOI: 10.1111/j.1654-109X.2012.01202.x

149. ORROCK, J.L., CHRISTOPHER, C.C. & DUTRA, H.P. (2012). Seed bank survival of an invasive species, but not of two native species, declines with invasion. *Oecologia* **168**, 1103–1110. DOI: 10.1007/s00442-011-2159-x

150. Otfinowski, R. & Kenkel, N.C. (2010). Covariance between disturbance and soil resources dictates the invasibility of northern fescue prairies. *Biological Invasions* **12**, 1349–1361. DOI: 10.1007/s10530-009-9551-z

151. Parker, I.M. (2001). Safe site and seed limitation in *Cytisus scoparius* (Scotch broom): Invasibility, disturbance, and the role of cryptogams in a glacial outwash prairie. *Biological Invasions* **3**, 323–332. DOI: 10.1023/A:1015855515361

152. Parker, I.M. & Gilbert, G.S. (2007). When there is no escape: the effects of natural enemies on native, invasive, and noninvasive plants. Ecology **88**, 1210–1224. DOI: 10.1890/06-1377

153. Pearson, D.E., Callaway, R.M. & Maron, J.L. (2011). Biotic resistance via granivory: establishment by invasive, naturalized, and native asters reflects generalist preference. *Ecology* **92**, 1748–1757. DOI: 10.1890/11-0164.1

154. Pearson, D.E., Hierro, J.L., Chiuffo, M. & Villarreal, D. (2014). Rodent seed predation as a biotic filter influencing exotic plant abundance and distribution. *Biological Invasions* **16**, 1185–1196. DOI: 10.1007/s10530-013-0573-1

155. Pearson, D.E., Potter, T. & Maron, J.L. (2012). Biotic resistance: Exclusion of native rodent consumers releases populations of a weak invader. *Journal of Ecology* 100, 1383–1390. DOI: 10.1111/j.1365-2745.2012.02025.x

156. Pearson, D.E., Icasatti, N.S., Hierro, J.L. & Bird, B.J. (2014). Are local filters blind to provenance? Ant seed predation suppresses exotic plants more than natives. *PLOS ONE* **9**, e110725. DOI: 10.1371/journal.pone.0103824

157. Peart, D.R. (1989). Species interactions in a successional grassland. III. Effects of canopy gaps, gopher mounds and grazing on colonization. *Journal of Ecology* **77**, 267–289. DOI: 10.2307/2260929

158. Perera, P.C.D., Szymura, T.H., de Patoul, L., Sladkovska, T. & Szymura, M. (2021). A community resembling semi-natural meadow is as resistant to goldenrod

invasion as highly productive commercial grassland. *Management of Biological Invasions* **12**, 873–885. DOI: 10.3391/MBI.2021.12.4.07

159. PETRUZZELLA, A., DA S. S. R. RODRIGUES, T.A., VAN LEEUWEN, C.H.A., DE ASSIS ESTEVES, F., FIGUEIREDO-BARROS, M.P. & BAKKER, E.S. (2020). Species identity and diversity effects on invasion resistance of tropical freshwater plant communities. *Scientific Reports* **10**, 5626. DOI: 10.1038/s41598-020-62660-1

160. PETRUZZELLA, A., MANSCHOT, J., VAN LEEUWEN, C.H.A., GRUTTERS, B.M.C. & BAKKER, E.S. (2018). Mechanisms of invasion resistance of aquatic plant communities. *Frontiers in Plant Science* **9**, 134. DOI: 10.3389/fpls.2018.00134

161. PIERSON, E.A. & MACK, R.N. (1990). The population biology of *Bromus tectorum* in forests: effect of disturbance, grazing, and litter on seedling establishment and reproduction. *Oecologia* 84, 526–533. DOI: 10.1007/BF00328170

162. PREVÉY, J.S., GERMINO, M.J. & HUNTLY, N.J. (2010). Loss of foundation species increases population growth of exotic forbs in sagebrush steppe. *Ecological Applications* **20**, 1890–1902. DOI: 10.1890/09-0750.1

163. PRIEUR-RICHARD, A.-H., LAVOREL, S., GRIGULIS, K. & DOS SANTOS, A. (2000). Plant community diversity and invasibility by exotics: invasion of Mediterranean old fields by *Conyza bonariensis* and *Conyza canadensis*. *Ecology Letters* **3**, 412–422. DOI: 10.1046/j.1461-0248.2000.00157.x

164. PROENÇA, B., NEZ, T., POLI, A., CIUTAT, A., DEVAUX, L., SOTTOLICHIO, A., DE MONTAUDOUIN, X. & MICHALET, R. (2019). Intraspecific facilitation explains the spread of the invasive engineer *Spartina anglica* in Atlantic salt marshes. *Journal of Vegetation Science* **30**, 212–223. DOI: 10.1111/jvs.12720

165. PUCHETA, E., GARCÍA-MURO, V.J., QUEVEDO-ROBLEDO, L. & ROLHAUSER, A. (2012). Winter establishment of the alien annual *Schismus barbatus* is not affected by insect

herbivory in Northern-Central Monte Desert. *Journal of Arid Environments* **79**, 120–123. DOI: 10.1016/j.jaridenv.2011.11.018

166. QIN, T.-J., ZHOU, J., SUN, Y., MÜLLER-SCHÄRER, H., LUO, F.-L., DONG, B.-C., LI, H.-L. & YU, F.-H. (2020). Phylogenetic diversity is a better predictor of wetland community resistance to *Alternanthera philoxeroides* invasion than species richness. *Plant Biology* **22**, 591–599. DOI: 10.1111/plb.13101
167. QUINN, L.D. & HOLT, J.S. (2009). Restoration for resistance to invasion by giant reed (*Arundo donax*). *Invasive Plant Science and Management* **2**, 279–291. DOI: 10.1614/IPSM-09-001.1
168. RADNY, J., VAN DER PUTTEN, W.H., TIELBÖRGER, K. & MEYER, K.M. (2018). Influence of seed size on performance of non-native annual plant species in a novel community at two planting densities. *Acta Oecologica* **92**, 131–137. DOI: 10.1016/j.actao.2018.05.005
169. RAFFAELE, E. (2004). Susceptibility of a Patagonian mallin flooded meadow to invasion by exotic species. *Biological Invasions* **6**, 473–481. DOI: 10.1023/B:BINV.0000041560.33770.97
170. RAUSCHERT, E.S.J. & SHEA, K. (2012). Influence of microsite disturbance on the establishment of two congeneric invasive thistles. *PLOS ONE* **7**, e45490. DOI: 10.1371/journal.pone.0045490
171. REINHART, K.O., PACKER, A., VAN DER PUTTEN, W.H. & CLAY, K. (2003). Plant-soil biota interactions and spatial distribution of black cherry in its native and invasive ranges. *Ecology Letters* **6**, 1046–1050. DOI: 10.1046/j.1461-0248.2003.00539.x
172. RICE, K.J. (1987). Interaction of disturbance patch size and herbivory in *Erodium* colonization. *Ecology* **68**, 1113–1115. DOI: 10.2307/1938386
173. RODRÍGUEZ, J., CALBI, M., ROILOA, S.R. & GONZÁLEZ, L. (2018). Herbivory induced non-local responses of the clonal invader *Carpobrotus edulis* are not mediated by clonal

integration. *Science of the Total Environment* **633**, 1041–1050. DOI: 10.1016/j.scitotenv.2018.03.264

174. SACCONE, P., GIREL, J., PAGES, J.-P., BRUN, J.-J. & MICHALET, R. (2013). Ecological resistance to *Acer negundo* invasion in a European riparian forest: relative importance of environmental and biotic drivers. *Applied Vegetation Science* **16**, 184–192. DOI: 10.1111/j.1654-109X.2012.01227.x

175. SACCONE, P., PAGES, J., GIREL, J., BRUN, J. & MICHALET, R. (2010). *Acer negundo* invasion along a successional gradient: early direct facilitation by native pioneers and late indirect facilitation by conspecifics. *New Phytologist* **187**, 831–842. DOI: 10.1111/j.1469-8137.2010.03289.x

176. SANDERS, N.J., WELTZIN, J.F., CRUTSINGER, G.M., FITZPATRICK, M.C., NUÑEZ, M.A., OSWALT, C.M. & LANE, K.E. (2007). Insects mediate the effects of propagule supply and resource availability on a plant invasion. *Ecology* **88**, 2383–2391. DOI: 10.1890/06-1449.1

177. SCHERBER, C., CRAWLEY, M.J. & POREMBSKI, S. (2003). The effects of herbivory and competition on the invasive alien plant *Senecio inaequidens* (Asteraceae). *Diversity and Distributions* **9**, 415–426. DOI: 10.1046/j.1472-4642.2003.00049.x

178. SCHIERENBECK, K.A., MACK, R.N. & SHARITZ, R.R. (1994). Effects of herbivory on growth and biomass allocation in native and introduced species of *Lonicera*. *Ecology* **75**, 1661–1672. DOI: 10.2307/1939626

179. SCHMIDT, R., DEISING, H.B., HENSEN, I., SCHÄDLER, M. & AUGE, H. (2020). Natural enemies do not contribute to negative frequency-dependence in native and exotic grassland plants. *Perspectives in Plant Ecology, Evolution and Systematics* **46**, 125565. DOI: 10.1016/j.ppees.2020.125565

180. SCHULTZ, E.L., ECKBERG, J.O., BERG, S.S., LOUDA, S.M. & MILLER, T.E.X. (2017). Native insect herbivory overwhelms context dependence to limit complex invasion dynamics of exotic weeds. *Ecology Letters* **20**, 1374–1384. DOI: 10.1111/ele.12833

181. SCHUSTER, M.J., WRAGG, P.D., ROTH, A.M., BOCKENSTEDT, P., FRELICH, L.E. & REICH, P.B. (2022). Using plants to control buckthorn (*Rhamnus cathartica*): improved biotic resistance of forests through revegetation. *Ecological Engineering* **182**, 106730. DOI: 10.1016/j.ecoleng.2022.106730

182. SEASTEDT, T.R. & SUDING, K.N. (2007). Biotic constraints on the invasion of diffuse knapweed (*Centaurea diffusa*) in North American grasslands. *Oecologia* **151**, 626–636. DOI: 10.1007/s00442-006-0620-z

183. SHAN, L. & HOU, M. (2024). Herbivore and native plant diversity synergistically resist alien plant invasion regardless of nutrient conditions. *Plant Diversity* 46, 640–647. DOI: 10.1016/j.pld.2023.09.002

184. SHANNON, S., FLORY, S.L. & REYNOLDS, H. (2012). Competitive context alters plant-soil feedback in an experimental woodland community. *Oecologia* **169**, 235–243. DOI: 10.1007/s00442-011-2195-6

185. SHAW, J.A., ROCHE, L.M., GORNISH, E.S., RAYBURN, A.P., ZAMORA, A.J. & LACA, E. (2023). Efficacy of strip seeding to restore grassland plant communities. *Restoration Ecology* **31**, e13822. DOI: 10.1111/rec.13822

186. SHELEY, R.L. & JAMES, J. (2010). Resistance of native plant functional groups to invasion by Medusahead (*Taeniatherum caput-medusae*). *Invasive Plant Science and Management* **3**, 294–300. DOI: 10.1614/IPSM-D-09-00056.1

187. SHEPPARD, C.S. & SCHURR, F.M. (2019). Biotic resistance or introduction bias? Immigrant plant performance decreases with residence times over millennia. *Global Ecology and Biogeography* **28**, 222–237. DOI: 10.1111/geb.12844

188. SILVA, A.D., ROCHE, L.M. & GORNISH, E.S. (2019). The use of strip-seeding for management of two late-season invasive plants. *Heliyon* **5**: e01772. DOI: 10.1016/j.heliyon.2019.e01772

189. SILVEIRA, M.J. & THOMAZ, S.M. (2023). Effects of interactions between abiotic and biotic factors on growth of a non-native macrophyte. *Biological Invasions* 25, 431–440. DOI: 10.1007/s10530-022-02924-1

190. SLATE, M.L., CALLAWAY, R.M. & PEARSON, D.E. (2019). Life in interstitial space: biocrusts inhibit exotic but not native plant establishment in semi-arid grasslands. *Journal of Ecology* **107**, 1317–1327. DOI: 10.1111/1365-2745.13117

191. SMITH, A.M., REINHARDT ADAMS, C., WIESE, C. & WILSON, S.B. (2016). Re-vegetation with native species does not control the invasive *Ruellia simplex* in a floodplain forest in Florida, USA. *Applied Vegetation Science* **19**, 20–30. DOI: 10.1111/avsc.12188

192. SMITH, M.D., WILCOX, J.C., KELLY, T. & KNAPP, A.K. (2004). Dominance not richness determines invasibility of tallgrass prairie. *Oikos* **106**, 253–262. DOI: 10.1111/j.0030-1299.2004.13057.x

193. SMULDERS, F.O.H., BECKER, S.T., CAMPBELL, J.E., BAKKER, E.S., BOÄSSON, M.J., BOUWMEESTER, M.M., VONK, J.A. & CHRISTIANEN, M.J.A. (2022). Fish grazing enhanced by nutrient enrichment may limit invasive seagrass expansion. *Aquatic Botany* **176**, 103464. DOI: 10.1016/j.aquabot.2021.103464

194. SONG, G., LI, X. & HUI, R. (2017). Biological soil crusts determine the germination and growth of two exotic plants. *Ecology and Evolution* **7**, 9441–9450. DOI: 10.1002/ece3.3477

195. SONG, G., LI, X. & HUI, R. (2020). Biological soil crusts increase stability and invasion resistance of desert revegetation communities in northern China. *Ecosphere* **11**, e03043. DOI: 10.1002/ecs2.3043

196. Sperfeld, E., Schmidtke, A., Gaedke, U. & Weithoff, G. (2010). Productivity, herbivory, and species traits rather than diversity influence invasibility of experimental phytoplankton communities. *Oecologia* **163**, 997–1010. DOI: 10.1007/s00442-010-1594-4

197. St. Clair, S.B. & Bishop, T.B.B. (2019). Loss of biotic resistance and high propagule pressure promote invasive grass-fire cycles. *Journal of Ecology* 107, 1995–2005. DOI: 10.1111/1365-2745.13156

198. Staab, K., Yannelli, F.A., Lang, M. & Kollmann, J. (2015). Bioengineering effectiveness of seed mixtures for road verges: Functional composition as a predictor of grassland diversity and invasion resistance. *Ecological Engineering* **84**, 104–112. DOI: 10.1016/j.ecoleng.2015.07.016

199. Steele, L.T., Ray, C. & Guidone, M. (2018). High phenolic content fails to deter mesograzer consumption of *Myriophyllum spicatum* (Eurasian watermilfoil) in New England. *Aquatic Ecology* **52**, 255–267. DOI: 10.1007/s10452-018-9661-8

200. Stricker, K.B. & Stiling, P. (2012). Herbivory by an introduced asian weevil negatively affects population growth of an invasive Brazilian shrub in Florida. *Ecology* **93**, 1902–1911. DOI: 10.1890/11-1328.1

201. Strong, JA, Maggs, CA & Johnson, MP (2009). The extent of grazing release from epiphytism for *Sargassum muticum* (Phaeophyceae) within the invaded range. *Journal of the Marine Biological Association of the United Kingdom* **89**, 303–314. DOI: 10.1017/S0025315408003226

202. Sun, Y., Müller-Schärer, H. & Schaffner, U. (2014). Plant neighbours rather than soil biota determine impact of an alien plant invader. *Functional Ecology* **28**, 1545–1555. DOI: 10.1111/1365-2435.12295

203. SUWA, T. & LOUDA, S.M. (2012). Combined effects of plant competition and insect herbivory hinder invasiveness of an introduced thistle. *Oecologia* 169, 467–476. DOI: 10.1007/s00442-011-2207-6

204. SUWA, T., LOUDA, S.M. & RUSSELL, F.L. (2010). No interaction between competition and herbivory in limiting introduced *Cirsium vulgare* rosette growth and reproduction. *Oecologia* 162, 91–102. DOI: 10.1007/s00442-009-1432-8

205. TE BEEST, M., MPANDZA, N.J. & OLFF, H. (2015). Fire and simulated herbivory have antagonistic effects on resistance of savanna grasslands to alien shrub invasion. *Journal of Vegetation Science* **26**, 114–122. DOI: 10.1111/jvs.12208

206. TE BEEST, M., MPANDZA, N.J. & OLFF, H. (2018). Biotic resistance affects growth and reproduction, but not survival of a high-impact woody invader in African savannas. *Journal of Vegetation Science* **29**, 532–540. DOI: 10.1111/jvs.12633

207. TECCO, P.A., DÍAZ, S., GURVICH, D.E., PEREZ-HARGUINDEGUY, N., CABIDO, M. & BERTONE, G.A. (2007). Facilitation and interference underlying the association between the woody invaders *Pyracantha angustifolia* and *Ligustrum lucidum*. *Applied Vegetation Science* **10**, 211–218. DOI: 10.1111/j.1654-109X.2007.tb00519.x

208. TEIXEIRA, M.C., BINI, L.M. & THOMAZ, S.M. (2017). Biotic resistance buffers the effects of nutrient enrichment on the success of a highly invasive aquatic plant. *Freshwater Biology* **62**, 65–71. DOI: 10.1111/fwb.12849

209. TERHORST, C.P. & LAU, J.A. (2015). Genetic variation in invasive species response to direct and indirect species interactions. *Biological Invasions* 17, 651–659. DOI: 10.1007/s10530-014-0756-4

210. TERHORST, C.P., WIRTH, C. & LAU, J.A. (2018). Genetic variation in mutualistic and antagonistic interactions in an invasive legume. *Oecologia* **188**, 159–171. DOI: 10.1007/s00442-018-4211-6

211. THEBAUD, C., FINZI, A.C., AFFRE, L., DEBUSSCHE, M. & ESCARRE, J. (1996). Assessing why two introduced *Conyza* differ in their ability to invade Mediterranean old fields. *Ecology* **77**, 791–804. DOI: 10.2307/2265502

212. THÉBAULT, A., STOLL, P. & BUTTLER, A. (2012). Complex interactions between spatial pattern of resident species and invasiveness of newly arriving species affect invasibility. *Oecologia* **170**, 1133–1142. DOI: 10.1007/s00442-012-2376-y

213. THIÉBAUT, G., TARAYRE, M. & RODRÍGUEZ-PÉREZ, H. (2019). Allelopathic effects of native versus invasive plants on one major invader. *Frontiers in Plant Science* **10**, 854. DOI: 10.3389/fpls.2019.00854

214. TIPPING, P.W., SMITH, M.C., LAKE, E.C., MINTEER, C.R., GOODE, A.B.C., FOLEY, J.R. & GETTYS, L.A. (2020). Classical biological control and apparent competition: Evaluating a waterhyacinth invaded community module. *Journal of Applied Ecology* **57**, 926–935. DOI: 10.1111/1365-2664.13593

215. UYÀ, M., BULLERI, F. & GRIBBEN, P.E. (2018). Propagules are not all equal: traits of vegetative fragments and disturbance regulate invasion success. *Ecology* **99**, 957–965. DOI: 10.1002/ecy.2168

216. VALLIERE, J.M. & ALLEN, E.B. (2016). Nitrogen enrichment contributes to positive responses to soil microbial communities in three invasive plant species. *Biological Invasions* **18**, 2349–2364. DOI: 10.1007/s10530-016-1166-6

217. VAN DER COLFF, D., DREYER, L.L., VALENTINE, A. & ROETS, F. (2017). Differences in physiological responses to infection by *Ceratocystis tsitsikammensis*, a native ophiostomatoid pathogen, between a native forest and an exotic forestry tree in South Africa. *Fungal Ecology* **27**, 107–115. DOI: 10.1016/j.funeco.2016.06.003

218. WALLER, L.P., CALLAWAY, R.M., KLIRONOMOS, J.N., ORTEGA, Y.K. & MARON, J.L. (2016). Reduced mycorrhizal responsiveness leads to increased competitive tolerance in

an invasive exotic plant. *Journal of Ecology* **104**, 1599–1607. DOI: 10.1111/1365-2745.12641

219. WANG, X.-Y., GAO, S., CHEN, T., WANG, J. & YU, F.-H. (2023). Interactions between soil microbes and native species drive a diversity-invasibility relationship. *Biological Invasions* 25, 1461–1472. DOI: 10.1007/s10530-022-02988-z

220. WANG, Y.-J., CHEN, D., YAN, R., YU, F.-H. & VAN KLEUNEN, M. (2019). Invasive alien clonal plants are competitively superior over co-occurring native clonal plants. *Perspectives in Plant Ecology, Evolution and Systematics* **40**, 125484. DOI: 10.1016/j.ppees.2019.125484

221. WEI, W., ZHU, P., CHEN, P., HUANG, Q., BAI, X., NI, G. & HOU, Y. (2020). Mixed evidence for plant–soil feedbacks in forest invasions. *Oecologia* **193**, 665–676. DOI: 10.1007/s00442-020-04703-y

222. WELLS, H.B.M., CREGO, R.D., ALSTON, J.M., NDUNG'U, S.K., KHASOHA, L.M., REED, C.G., HASSAN, A.A., KURUKURA, S., EKADELI, J., NAMONI, M., STEWART, P.S., KIMUYU, D.M., WOLF, A.A., YOUNG, T.P., KARTZINEL, T.R., PALMER, T.M., GOHEEN, J.R. & PRINGLE, R.M. (2023). Wild herbivores enhance resistance to invasion by exotic cacti in an African savanna. *Journal of Ecology* **111**, 33–44. DOI: 10.1111/1365-2745.14010

223. WHITE, T.A., CAMPBELL, B.D., KEMP, P.D. & HUNT, C.L. (2001). Impacts of extreme climatic events on competition during grassland invasions. *Global Change Biology* **7**, 1–13. DOI: 10.1046/j.1365-2486.2001.00381.x

224. WHITFELD, T.J.S., ROTH, A.M., LODGE, A.G., EISENHAUER, N., FRELICH, L.E. & REICH, P.B. (2014). Resident plant diversity and introduced earthworms have contrasting effects on the success of invasive plants. *Biological Invasions* 16, 2181–2193. DOI: 10.1007/s10530-014-0657-6

225. WILLIAMSON, J. & HARRISON, S. (2002). Biotic and abiotic limits to the spread of exotic revegetation species. *Ecological Applications* **12**, 40–51. DOI: 10.2307/3061135

226. WILSEY, B., KAUL, A. & POLLEY, H.W. (2024). Establishment from seed is more important for exotic than for native plant species. *Plant-Environment Interactions* **5**, e10132. DOI: 10.1002/pei3.10132

227. XIE, D., YU, D., YOU, W.-H. & XIA, C.-X. (2013). The propagule supply, litter layers and canopy shade in the littoral community influence the establishment and growth of *Myriophyllum aquaticum*. *Biological Invasions* **15**, 113–123. DOI: 10.1007/s10530-012-0272-3

228. XU, K., YE, W., CAO, H., DENG, X., YANG, Q. & ZHANG, Y. (2004). The role of diversity and functional traits of species in community invasibility. *Botanical Bulletin of Academia Sinica* **45**, 149–157. DOI: 10.1614/0890-037X(2004)018[1301:TROSTI]2.0.CO,2

229. XU, X., ZHANG, Y., LI, S., CHEN, H., LIU, M., LI, B. & NIE, M. (2022). Native herbivores indirectly facilitate the growth of invasive *Spartina* in a eutrophic saltmarsh. *Ecology* **103**, e3610. DOI: 10.1002/ecy.3610

230. XUE, W., YAO, S.-M., HUANG, L., ROILOA, S.R., JI, B.-M. & YU, F.-H. (2022). Current plant diversity but not its soil legacy influences exotic plant invasion. *Journal of Plant Ecology* **15**, 639–649. DOI: 10.1093/jpe/rtab065

231. YANNELLI, F.A., KARRER, G., HALL, R., KOLLMANN, J. & HEGER, T. (2018). Seed density is more effective than multi-trait limiting similarity in controlling grassland resistance against plant invasions in mesocosms. *Applied Vegetation Science* **21**, 411–418. DOI: 10.1111/avsc.12373

232. YANNELLI, F.A., MACLAREN, C. & KOLLMANN, J. (2020). Moving away from limiting similarity during restoration: timing of arrival and native biomass are better proxies of invasion suppression in grassland communities. *Frontiers in Ecology and Evolution* **8**, 238. DOI: 10.3389/fevo.2020.00238

233. YU, N.X., LI, C.W., ZHU, P., ZHANG, J.L., ZHANG, P. & HOU, Y.P. (2019). Allelopathic effects of *Robinia pseudoacacia* and *Quercus acutissima* on the exotic plant *Rhus*

typhina in Shandong peninsula. *Allelopathy Journal* **47**, 181–193. DOI: 10.26651/allelo.j/2019-47-2-1230

234. YUAN, L., LI, J. & VAN KLEUNEN, M. (2022). Diversity of resident plant communities could weaken their allelopathic resistance against alien and native invaders. *Biological Invasions* **24**, 607–619. DOI: 10.1007/s10530-021-02667-5

235. ZAVALETA, E.S. & HULVEY, K.B. (2004). Realistic species losses disproportionately reduce grassland resistance to biological invaders. *Science* **306**, 1175–1177. DOI: 10.1126/science.1102643

236. ZAVALETA, E.S. & HULVEY, K.B. (2007). Realistic variation in species composition affects grassland production, resource use and invasion resistance. *Plant Ecology* **188**, 39–51. DOI: 10.1007/s11258-006-9146-z

237. ZHANG, Y., MENG, H., WANG, Y. & HE, Q. (2018). Herbivory enhances the resistance of mangrove forest to cordgrass invasion. *Ecology* **99**, 1382–1390. DOI: 10.1002/ecy.2233

238. ZHANG, Z., LIU, Y., BRUNEL, C. & VAN KLEUNEN, M. (2020). Evidence for Elton's diversity–invasibility hypothesis from belowground. *Ecology* **101**, e03187. DOI: 10.1002/ecy.3187

239. ZHAO, Y.Z., LIU, M.C., FENG, Y.L., WANG, D., FENG, W.W., CLAY, K., DURDEN, L.A., LU, X.R., WANG, S., WEI, X.L. & KONG, D.L. (2020). Release from below- and aboveground natural enemies contributes to invasion success of a temperate invader. *Plant and Soil* **452**, 19–28. DOI: 10.1007/s11104-020-04520-5

240. ZHENG, Y.-L., BURNS, J.H., LIAO, Z.-Y., LI, Y.-P., YANG, J., CHEN, Y.-J., ZHANG, J.-L. & ZHENG, Y.-G. (2018). Species composition, functional and phylogenetic distances correlate with success of invasive *Chromolaena odorata* in an experimental test. *Ecology Letters* 21, 1211–1220. DOI: 10.1111/ele.13090

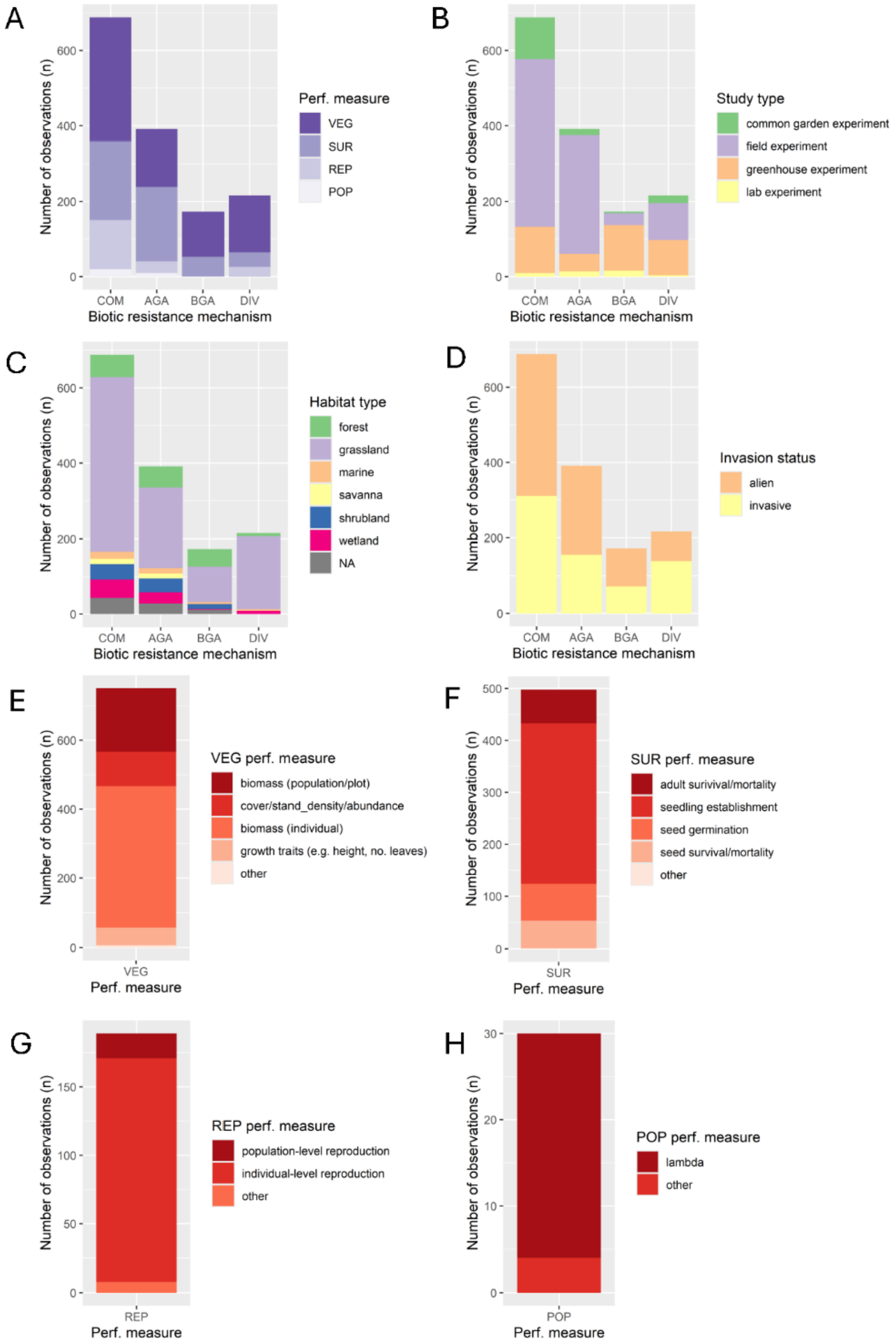

Fig. S1. Distribution of the 1468 observations from 240 studies on alien plant performance according to biotic resistance mechanism, performance measure and various moderators. (A) Distribution of performance (perf.) measure (VEG = vegetative performance, SUR = survival, REP = reproductive performance and POP = population growth) within each of the four biotic

resistance mechanisms (COM = competition, AGA = aboveground antagonism, BGA = belowground antagonism and DIV = diversity–invasibility); (B–D) Distribution of the various study types, habitat types and invasion status within each of the four biotic resistance mechanisms; and (E–H) Distribution of the specific measures within each performance measure category. Note here that some studies may have included multiple performance measures, in which case only one was chosen per performance category, with order of preference consistent with the order of categories in the legend from top to bottom (e.g. "lambda" was preferred over "other" in the population growth category).

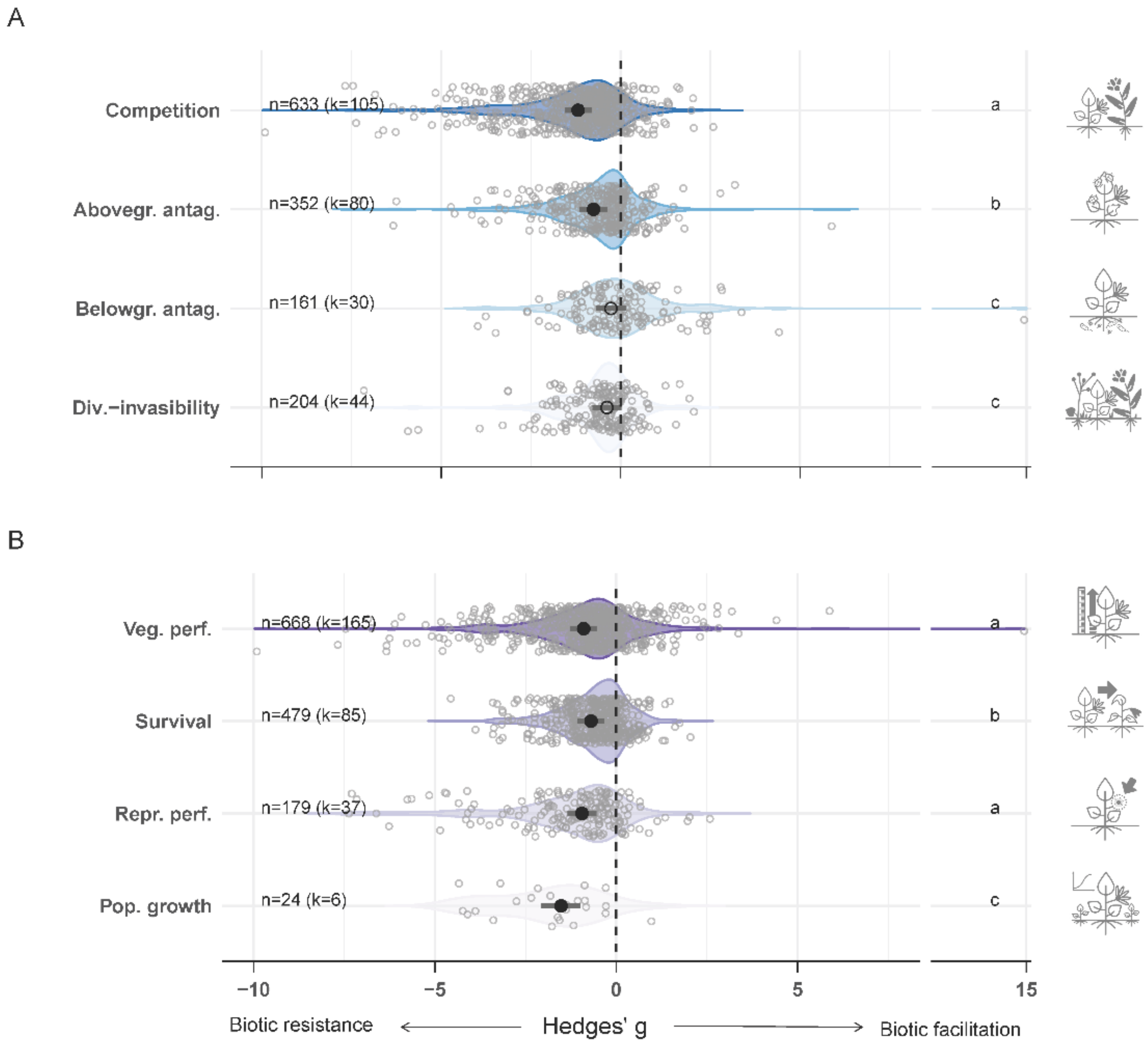

Fig. S2. Results of a phylogenetic model without an interaction between biotic resistance mechanism and performance measure (given that a phylogenetic model including the interaction was only marginally significantly better than one without). Effects of **A** biotic resistance mechanism and **B** plant performance measure on Hedges' *g* (magnitude and direction of biotic resistance). The overall adjusted mean Hedges' *g* of this model was -0.84 and a 95% confidence interval ranging between -1.2 and -0.48 (total I^2 = 97.6 %; among studies: 35.1%; among groups: 16.9%; among observations: 38.0%; phylogenetic relationships: 7.6 %; marginal R^2 = 17.0%, conditional R^2 = 67.7%). Negative values indicate biotic resistance (i.e. lower performance in the presence/high diversity of native species). Biotic resistance mechanism ($F_{3,1343}$ = 28.1, P < 0.001) and performance measure ($F_{3,1343}$ = 7.7, P <

0.001) each had highly significant effects. The plot shows predicted effect sizes (black filled circles for significant effects, black open circles for non-significant effects) with 95% confidence intervals (dark grey lines) and the distribution of effect sizes (in colour). Different letters on the right show significant differences between treatment levels. Competition provided the strongest biotic resistance, followed by aboveground antagonism. Population growth was most strongly reduced by native species, survival the least. Pairwise differences were always significant, except for effects of diversity–invasibility not differing from effects of belowground antagonism, and vegetative and reproductive performance being equally strongly negatively affected. Sample sizes are given on the left as the number of observations (*n*) with the number of studies (*k*) in parentheses. Note the break in the x-axis to enhance visibility.

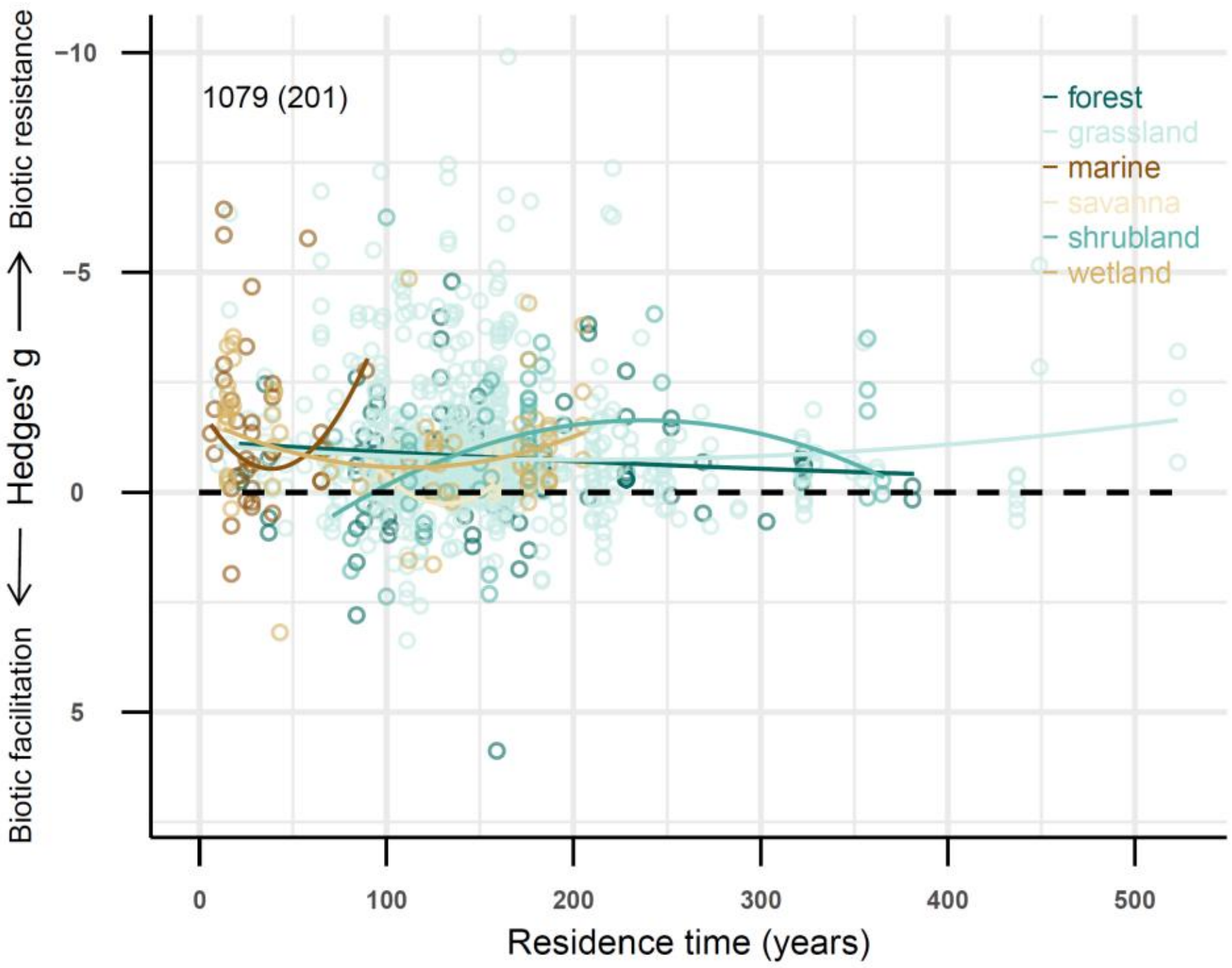

Fig. S3. Effect of residence time of alien plant species on the magnitude of biotic resistance (Hedges' *g*) to plant invasions. Negative values indicate biotic resistance (i.e. lower performance in the presence/high diversity of native species). Note that the y-axis is plotted on an inverted scale and that for better visibility, an outlier (one data point at residence time = 100 years and Hedges' *g* = 14.9) was not plotted. Sample size is given on the left as the number of observations (*n*) with the number of studies (*k*) in parentheses. Relationships between residence time and Hedges' *g* are shown for 6 different categories of habitat type: dark teal = forest, medium teal = shrubland, light teal = grassland, dark brown = marine, medium brown = wetland, light brown = savanna (prediction lines are only shown within the range of residence time data for each category).

Table S1. List of most common alien species (i.e. occurring in ≥ 5 of the 240 included studies in the meta-analysis). 59 % of studies focused on 1 alien species, 3 % of studies showed only averaged results across multiple species, and 30 % of studies focused on species-specific effects of 2 or more alien species.

**Species**	**Number of studies**
Centaurea solstitialis	15
Bromus tectorum	13
Centaurea stoebe	9
Solidago canadensis	9
Alternanthera philoxeroides	7
Bidens pilosa	6
Cirsium vulgare	6
Lolium perenne	6
Senecio inaequidens	6
Erigeron canadensis	5
Lolium multiflorum	5
Tragopogon dubius	5

Table S2. Meta-analysis models, shown with their significance tests (*F* tests for factors, standardised effect sizes *t* for continuous variables); significant values ($P < 0.05$) are highlighted in bold font, marginally significant values ($P < 0.1$) in italics), amount of explained variation (R^2), heterogeneity between effect sizes (I^2) and number of observations (*n*). Note that each model included the interaction of biotic resistance (BR) mechanism and performance (perf.) measure. The residence time model additionally considered main effects of (linear and quadratic) log-transformed minimum residence time (MRT). The models with other moderators included the main effect of one single extra moderator, except for the model testing for time-lag and publication bias, which included both. The alien *vs*. native model additionally included data on native species and tested for interactions with species origin. [1] = Given that the study design is not fully factorial (i.e. some combinations of categorical predictors are missing), the test cannot estimate degrees of freedom correctly and thus pools those that cannot be assigned to either the main effects of interaction in a single category, here called "confounded". [2] = Given the marginal significance, results of the model without interaction are

reported in Fig. S2. [3] = Factor with 6 levels: forest, shrubland, savanna, grassland, wetland, marine. [4] = Factor with 2 levels: terrestrial *vs*. aquatic. [5] = Factor with 2 levels: non-invasive alien *vs*. invasive.

**Research question**	**Model**	**Fixed effects**	**Significance**	**R^2 marginal (conditional) (%)**	**I^2 total (study, group, observation, phylogeny) (%)**	***n***
1	Main model	BR mechanism<br>perf. measure<br>BR mechanism x perf. measure<br>Confounded[1]	**$F_{1,1422}$=27.6, P<0.001**<br>**$F_{1,1422}$=6.9, P=0.009**<br>**$F_{6,1422}$=2.6, P=0.016**<br>**$F_{4,1422}$=18.1, P<0.001**	19.8 (66.1)	97.5 (38.0, 18.3, 41.2)	1423
	Phylogenetic model	BR mechanism<br>perf. measure<br>BR mechanism x perf. measure<br>Confounded[1]	**$F_{1,1349}$=23.0, P<0.001**<br>**$F_{1,1349}$=5.4, P=0.020**<br>*$F_{6,1349}$=2.1, P=0.053*[2]<br>**$F_{4,1347}$=19.2, P<0.001**	19.0 (67.9)	97.6 (34.4, 16.4, 38.6, 8.1)	1350
	Model to test for time-lag and publication bias	BR mechanism<br>Perf. measure<br>BR mechanism x perf. measure<br>Confounded[1]<br>Time-lag bias<br>Publication bias	**$F_{1,931}$=21.6, P<0.001**<br>NA<br>$F_{3,931}$=0.9, P=0.454<br>**$F_{4,931}$=10.7, P<0.001**<br>*t=1.936, P=0.053*<br>t=-1.032, P=0.302	21.9 (74.4)	78.8 (29.9, 23.1, 25.9)	932
3	Residence time model	BR mechanism<br>Perf. measure<br>BR mechanism x Perf. measure<br>Confounded[1]<br>MRT<br>Quadratic MRT	**$F_{1,1078}$=15.6, P<0.001**<br>**$F_{1,1078}$=11.2, P<0.001**<br>**$F_{6,1078}$=2.4, P=0.026**<br>**$F_{4,1078}$=18.5, P<0.001**<br>t=1.447, P=0.148<br>*t=-1.953, P=0.051*	23.4 (69.5)	97.6 (37.8, 21.0, 38.8)	1079
	Model with study type	BR mechanism<br>Perf. measure<br>BR mechanism x perf. measure<br>Study type<br>Confounded[1]	**$F_{1,1422}$=25.4, P<0.001**<br>**$F_{1,1422}$=4.1, P=0.043**<br>**$F_{6,1422}$=3.0, P=0.007**<br>$F_{3,1422}$=1.9, P=0.122<br>**$F_{4,1422}$=18.1, P<0.001**	21.1 (66.6)	97.5 (37.8, 18.5, 41.3)	1423
	Model with habitat type narrow	BR mechanism<br>Perf. measure<br>BR mechanism x perf. measure<br>Habitat type narrow[3]<br>Confounded[1]	**$F_{1,1343}$=27.3, P<0.001**<br>**$F_{1,1343}$=7.4, P=0.007**<br>**$F_{6,1343}$=2.6, P=0.015**<br>$F_{5,1343}$=0.6, P=0.699<br>**$F_{4,1343}$=18.2, P<0.001**	21.6 (66.2)	97.4 (36.6, 18.8, 42.0)	1344
	Model with habitat type broad	BR mechanism<br>Perf. measure<br>BR mechanism x perf. measure<br>Habitat type broad[4]<br>Confounded[1]	**$F_{1,1416}$=27.0, P<0.001**<br>**$F_{1,1416}$=6.0, P=0.015**<br>**$F_{6,1416}$=2.7, P=0.014**<br>$F_{1,1416}$=1.8, P=0.180<br>**$F_{4,1416}$=18.0, P<0.001**	20.0 (65.4)	97.5 (37.0, 18.4, 42.1)	1417

	Model with spatial scale	BR mechanism<br>Perf. measure<br>BR mechanism x perf. measure<br>Confounded[1]<br>Log spatial scale	**$F_{1,1368}$=27.1, P<0.001**<br>**$F_{1,1368}$=10.3, P=0.001**<br>**$F_{6,1368}$=2.2, P=0.039**<br><br>**$F_{4,1368}$=16.9, P<0.001**<br>t=-0.144, P=0.885	19.5 (68.9)	97.5 (38.3, 21.6, 37.6)	1369
	Model with temporal scale	BR mechanism<br>Perf. measure<br>BR mechanism x perf. measure<br>Confounded[1]<br>Log temporal scale	**$F_{1,1344}$=24.9, P<0.001**<br>**$F_{1,1344}$=7.4, P=0.007**<br>**$F_{6,1344}$=2.7, P=0.012**<br><br>**$F_{4,1344}$=16.1, P<0.001**<br>**t=3.541, P<0.001**	20.9 (63.6)	96.5 (30.6, 21.5, 44.4)	1345
	Model with invasion status	BR mechanism<br>Perf. measure<br>BR mechanism x perf. measure<br>Invasion status[5]<br>Confounded[1]	**$F_{1,1422}$=27.6, P<0.001**<br>**$F_{1,1422}$=5.7, P=0.019**<br>**$F_{6,1422}$=2.8, P=0.011**<br><br>**$F_{1,1422}$=4.0, P=0.045**<br>**$F_{4,1422}$=18.1, P<0.001**	20.5 (66.1)	97.5 (37.5, 18.4, 41.5)	1423
4	Alien *vs.* native model	BR mechanism<br>Perf. measure<br>Origin<br>BR mechanism x perf. measure<br>BR mechanism x origin<br>Perf. measure x origin<br>Confounded[1]	**$F_{1,912}$=16.1, P<0.001**<br>**$F_{1,912}$=9.3, P=0.002**<br>$F_{1,912}$=2.0, P=0.163<br>**$F_{4,912}$=2.6, P=0.035**<br><br>$F_{3,912}$=1.5, P=0.225<br><br>$F_{2,912}$=1.7, P=0.180<br><br>**$F_{3,912}$=12.3, P<0.001**	22.7 (62.1)	95.1 (36.5, 12.0, 46.6)	913
	Alien *vs.* native phylogenetic model	BR mechanism[1]<br>Perf. measure<br>Origin<br>BR mechanism x perf. measure<br>BR mechanism x origin<br>Perf. measure x origin<br>Confounded[1]	NA<br>**$F_{1,840}$=8.0, P=0.005**<br>$F_{1,840}$=1.8, P=0.179<br>**$F_{6,840}$=4.5, P=0.004**<br><br>$F_{3,840}$=1.0, P=0.375<br><br>$F_{2,840}$=1.4, P=0.246<br>**$F_{4,840}$=13.4, P<0.001**	23.3 (62.8)	94.7 (39.1, 9.7, 45.9, 0.0)	841

Table S3. Marginal means for individual moderators in the meta-analysis, calculated with the R package 'emmeans' (Lenth, 2024), providing proportions weights. Marginal means with their 95% confidence intervals are shown for the main model, phylogenetic model and alien *vs.* native model, with means for each combination of biotic resistance (BR) mechanism, performance (perf.) measure, and species origin.

Model	BR mechanism	Perf. measure	Origin	Marginal mean	95% confidence interval
Main model	Competition	Vegetative perf.		-1.218	-1.375, -1.062
		Survival		-0.886	-1.046, -0.726
		Rep. perf.		-1.261	-1.495, -1.027
		Pop. growth		-1.997	-2.462, -1.532
	Aboveground antagonism	Vegetative perf.		-0.685	-0.886, -0.484
		Survival		-0.588	-0.800, -0.376
		Rep. perf.		-0.606	-1.001, -0.212
		Pop. growth		- 0.290	-1.009, 0.429
	Belowground antagonism	Vegetative perf.		-0.132	-0.403, 0.139
		Survival		-0.187	-0.515, 0.141
		Rep. perf.		NA	NA
		Pop. growth		NA	NA
	Diversity–invasibility	Vegetative perf.		-0.320	-0.527, -0.113
		Survival		-0.262	-0.553, 0.029
		Rep. perf.		-0.397	-0.795, 0.000
		Pop. growth		NA	NA
Phylogenetic model	Competition	Vegetative perf.		-1.312	-1.705, -0.918
		Survival		-0.979	-1.370, -0.588
		Rep. perf.		-1.336	-1.764, -0.908
		Pop. growth		-2.086	-2.673, -1.499
	Aboveground antagonism	Vegetative perf.		-0.766	-1.180, -0.351
		Survival		-0.706	-1.116, -0.296
		Rep. perf.		-0.780	-1.308, -0.251
		Pop. growth		-0.652	-1.505, 0.200
	Belowground antagonism	Vegetative perf.		-0.211	-0.664, 0.242
		Survival		-0.282	-0.770, 0.205
		Rep. perf.		NA	NA
		Pop. growth		NA	NA
	Diversity–invasibility	Vegetative perf.		-0.378	-0.796, 0.040
		Survival		-0.359	-0.825, 0.106
		Rep. perf.		-0.466	-1.004, 0.071
		Pop. growth		NA	NA
Alien *vs*. native model	Competition	Vegetative perf.	Alien	-0.957	-1.194, -0.721
			Native	-0.709	-0.939, -0.478
		Survival	Alien	-0.804	-1.011, -0.598
			Native	-0.557	-0.761, -0.353
		Rep. perf.	Alien	-0.911	-1.365, -0.456
			Native	-1.167	-1.692, -0.642
		Pop. growth	Alien	NA	NA
			Native	NA	NA
	Aboveground antagonism	Vegetative perf.	Alien	-0.604	-0.843, -0.365
			Native	-0.551	-0.788, -0.314
		Survival	Alien	-0.188	-0.408, 0.032
			Native	-0.136	-0.356, 0.084
		Rep. perf.	Alien	-0.527	-1.586, 0.533
			Native	-0.979	-2.049, 0.091
		Pop. growth	Alien	NA	NA
			Native	NA	NA
	Belowground antagonism	Vegetative perf.	Alien	-0.325	-0.764, 0.115
			Native	-0.350	-0.827, 0.127

		Survival	Alien	0.133	-0.162, 0.438
			Native	0.107	-0.183, 0.396
		Rep. perf.	Alien	NA	NA
			Native	NA	NA
		Pop. growth	Alien	NA	NA
			Native	NA	NA
	Diversity–invasibility	Vegetative perf.	Alien	-0.125	-0.550, 0.301
			Native	-0.107	-0.505, 0.291
		Survival	Alien	-0.467	-0.843, -0.091
			Native	-0.451	-0.811, -0.091
		Rep. perf.	Alien	NA	NA
			Native	NA	NA
		Pop. growth	Alien	NA	NA
			Native	NA	NA

Table S4. The biotic resistance (BR) mechanism combinations with the numbers of effect sizes (number of observations, *n*) with additive, synergistic, antagonistic and reversal effects for each combination (continuous performance measures only). The 16 observations on survival which could not be included in the model are also listed.

BR mechanisms combination	***n* Additive**	***n* Synergistic**	***n* Antagonistic**	***n* Reversal**	***n* Survival**
Competition – aboveground antagonism	21	1	9	3	11
Competition – belowground antagonism				1	
Aboveground antagonism – belowground antagonism	1			2	3
Aboveground antagonism – diversity–invasibility	16		2	2	2
Belowground antagonism-diversity–invasibility	5	2			

Table S5. Effect of residence time depending on other moderators. Maximum likelihood ratio tests with degrees of freedom (*df*) and *P*-values (significant values in bold), testing whether models including an interaction between a given moderator (biotic resistance (BR) mechanism, performance (perf.) measure, or other moderators) and minimum residence time (MRT, linear and quadratic effects) is better compared to a model without that interaction.

**Interaction tested**	**Likelihood ratio test**
BR mechanism x MRT	X^2 (6 *df*) = 3.0, *P* = 0.805
Perf. measure x MRT	X^2 (6 *df*) = 9.7, *P* = 0.138
Study type x MRT	X^2 (6 *df*) = 5.7, *P* = 0.453

Habitat type narrow x MRT	**X^2 (10 *df*) = 20.8, *P* = 0.022**
Habitat type broad x MRT	X^2 (2 *df*) = 0.8, *P* = 0.655
Log spatial scale x MRT	X^2 (2 *df*) = 3.8, *P* = 0.151
Log temporal scale x MRT	X^2 (2 *df*) = 3.6, *P* = 0.163
Invasion status x MRT	X^2 (2 *df*) = 1.8, *P* = 0.407

Appendix S2. A mathematical model for the temporal dynamics of biotic resistance by native antagonists

Initialization of the model

We considered z = 30 distinct types of both invaders and native antagonists (indexed by i = 0..(z-1)). The initial density I_i of invader type i with intrinsic population growth rate $r_i = i/(z-1)$ was set to

$I_i(0) = I_{init}$ BetaBinomial(i, n = z-1, $\alpha = s_I$, $\beta = s_I$),

where I_{init} is the summed initial density of all invaders, BetaBinomial is the probability density function of the beta-binomial distribution with size parameter n and shape parameters α and β. We chose the beta-binomial distribution since it is a discrete, bounded distribution with adjustable variance. To ensure that the distribution of r is symmetric about its mean $\mu_r = 0.5$, we set $\alpha = \beta = s_I$, and to ensure that r has initial variance V_I we calculated s_I as

$s_I = (z-1)(1 - 4 V_I) / (8 (z-1) V_I - 2)$

For examples of initial frequency distributions of invader types with different r_i see Fig. 7A. Accordingly, the initial density N_j of native antagonist type j with attack rate $a_j = j/(z-1)$ was set to:

$N_j(0) = N_{init}$ BetaBinomial(i, n = z-1, $\alpha = s_N$, $\beta = s_N$),

with N_{init} (= 0.1) being the summed density of all antagonists and

$s_N = (z-1)(1 - 4 V_N) / (8 (z-1) V_N - 2)$,

which ensures that a has initial variance V_N and yields initial frequency distributions of native antagonists with different a_j shown in Fig. 7B.

Model parameterization

Model parameters that were kept constant in all scenarios are listed in Table S6.

Table S6. Model parameters that were kept constant in all scenarios.

Parameter	Symbol	Value
Carrying capacity of invaders	K	2
Conversion rate of consumed invader biomass into biomass of native antagonists	c	0.1
Mortality rate of native antagonists	m	0.1
Initial density of native antagonists	I_{init}	0.1

Solving the model

The system of ordinary differential equations defined by equations 1 and 2 was solved using the FORTRAN function lsoda (Petzold, 1983) accessed via R package deSolve (Soetaert *et al.,* 2010).

Measuring invasiveness and invasibility

In the model, the invasiveness of an invader type increases with its intrinsic population growth rate r_i. Hence, we measured overall invasiveness as the density-weighted mean population growth rate

$$\bar{r} = \sum_i r_i I_i / \sum_i I_i$$

Invasibility, on the other hand, decreases with the attack rate of the native antagonists community members. Hence, we measured overall invasibility as

$$(1 - \bar{a}) = 1 - \sum_j a_j N_j / \sum_j N_j$$

By subtracting $\bar{a}$ from 1 we ensured that the measures of invasibility ranges from 0 (minimal invasibility) to 1 (maximum invasibility) and thus in the same range as the measure of invasiveness, $\bar{r}$.

Measuring and decomposing biotic resistance

To measure total biotic resistance and decompose it into its determinants, we contrasted the temporal dynamics of overall invader density $I(t) = \sum I_i(t)$ obtained for alternative scenarios (Table S7). These scenarios differ in σ^2_r, σ^2_a and/or initial antagonist density N_{init}. For each scenario, we measured biotic resistance at time t as

$BR_{total}(t) = [I_{max}(t) - I(t)] / I_{max}(t)$,

where I is overall invader density in the respective scenario and I_{max} is overall invader density without biotic resistance (scenario 1 in Table S7), i.e. in the absence of native antagonists (N_{init} = 0) and with maximal initial variance in population growth rate of the invader (V_I = 0.08).

Table S7. Alternative scenarios for biotic resistance and corresponding parameter settings. Scenarios are ordered from no biotic resistance (absence of native antagonists) to maximum biotic resistance (full inter- and intraspecific variation in native antagonists, no variation in invaders). Comparisons among subsequent scenarios quantify the effect of different components of biotic resistance. The parameterization assumes that 75% of total trait variance (here 0.08) resides between species.

No	Native antagonists	Invaders	N_{init}	V_N	V_I	Comparison to scenario above measures effect of…
1	Absent	Inter- and intra-specific variation	0	0	0.08	-
2	No variation	Inter- and intra-specific variation	0.1	0	0.08	Native community density
3	Intraspecific variation	Inter- and intra-specific variation	0.1	0.02	0.08	Intraspecific variation in native community
4	Inter- and intra-specific variation	Inter- and intra-specific variation	0.1	0.08	0.08	Interspecific variation in native community
5	Inter- and intra-specific variation	Interspecific variation	0.1	0.08	0.06	Intraspecific variation in invaders
6	Inter- and intra-specific variation	No variation	0.1	0.08	0	Interspecific variation in invaders

References

LENTH, R. (2024) emmeans: Estimated marginal means, aka least-squares means. R package version 1.10.3, https://CRAN.R-project.org/package=emmeans.

PETZOLD, LR. (1983) Automatic Selection of Methods for Solving Stiff and Nonstiff Systems of Ordinary Differential Equations. *Siam Journal on Scientific & Statistical Computing* **4**, 136–148.

SOETAERT, K., PETZOLDT, T. & SETZER, R.W. (2010). Solving Differential Equations in R: Package deSolve. *Journal of Statistical Software*, **3**, 1–25.
